# Another unit Burr XII quantile regression model based on the different reparameterization applied to dropout in Brazilian undergraduate courses

**DOI:** 10.1371/journal.pone.0276695

**Published:** 2022-11-03

**Authors:** Tatiane Fontana Ribeiro, Fernando A. Peña-Ramírez, Renata Rojas Guerra, Gauss M. Cordeiro

**Affiliations:** 1 Instituto de Matemática e Estatística, Universidade de São Paulo, São Paulo, SP, Brazil; 2 Departamento de Estadística, Universidad Nacional de Colombia, Bogotá, Colombia; 3 Departamento de Estatística, Universidade Federal de Santa Maria, Santa Maria, RS, Brazil; 4 Departamento de Estatística, Universidade Federal de Pernambuco, Recife, PE, Brazil; University of Sargodha, PAKISTAN

## Abstract

In many practical situations, there is an interest in modeling bounded random variables in the interval (0, 1), such as rates, proportions, and indexes. It is important to provide new continuous models to deal with the uncertainty involved by variables of this type. This paper proposes a new quantile regression model based on an alternative parameterization of the unit Burr XII (UBXII) distribution. For the UBXII distribution and its associated regression, we obtain score functions and observed information matrices. We use the maximum likelihood method to estimate the parameters of the regression model, and conduct a Monte Carlo study to evaluate the performance of its estimates in samples of finite size. Furthermore, we present general diagnostic analysis and model selection techniques for the regression model. We empirically show its importance and flexibility through an application to an actual data set, in which the dropout proportion of Brazilian undergraduate animal sciences courses is analyzed. We use a statistical learning method for comparing the proposed model with the beta, Kumaraswamy, and unit-Weibull regressions. The results show that the UBXII regression provides the best fit and the most accurate predictions. Therefore, it is a valuable alternative and competitive to the well-known regressions for modeling double-bounded variables in the unit interval.

## 1 Introduction

University dropout is a problem with academic, social, and economic implications due to the high cost it inflicts on the students, their families, universities, and the country’s growth [1]. Thus, it is necessary to extract relevant information to enable higher education institutions (HEIs) to understand this phenomenon and minimize the dropout proportion of their courses. In that idea, several authors studied how aspects of the organizational structure of universities affect student outcomes. See [2, 3], for instance. However, it is essential to look at appropriate classes of regressions to model the dropout proportion, such as those based on distributions that lie on the standard unit interval.

The Beta [4] and Kumaraswamy [5, 6] regressions are the most widely used for modeling unit outcomes. The beta regression is useful to understand the influence of covariates on the response’s mean. The Kumaraswamy is the classical alternative to the beta and allows modeling the quantile of a response in the unit interval. However, the search for alternative unit regressions has attracted many researchers’ attention, especially those based on quantile approaches. For example, [7] introduced the unit-Weibull quantile regression. [8, 9] proposed the unit Burr XII and reflected unit Burr XII, respectively. Other quantile regressions were introduced by [10, 11], and [12]. One may also see [13–16] for unit regressions applied to educational measurements. These authors focus on comparing indicators from different countries, including educational attainment percentage, and school living conditions. However, to the author’s knowledge, there is still a lack of information concerning the phenomenon of student dropout.

Under the above information, the goal of this paper is to propose a new alternative for unit quantile regression applied to the dropout proportion of undergraduate courses. We use an approach based on the unit Burr XII (UBXII) distribution, which was pioneered [8] by applying the transformation method in a Burr XII (BXII) random variable. Their choice was based on the versatility of the baseline, which has been applied in reliability analysis [17], regression modeling [18], generalized distributions [19, 20], and several other disciplines. Let *Y* be a unit random variable having the UBXII distribution. The cumulative distribution function (cdf) and probability density function (pdf) of *Y* are

$$\begin{array}{c} F_{Y}(y;c,d)={\left(1+{\text{log}}^{c}y^{-1}\right)}^{-d},\;0<y<1, \end{array}$$ (1)

and

$$\begin{array}{c} f_{Y}(y;c,d)=c\;d\;y^{-1}{\text{log}}^{c-1}y^{-1}{\left(1+{\text{log}}^{c}y^{-1}\right)}^{-(d+1)}, \end{array}$$ (2)

respectively, where *c* > 0 and *d* > 0 are shape parameters. The quantile function (qf) of *Y* follows by inverting Eq (1), namely

$$\begin{array}{c} Q_{Y}(\tau )=\text{exp}\;\left[\;-{\left(\tau^{-1/d}-1\right)}^{1/c}\;\right],\;0<\tau <1. \end{array}$$ (3)

Henceforth, if *Y* is a random variable with pdf (2), we write *Y*∼ UBXII (*c*, *d*). For *c* = 1, the UBXII distribution reduces to the unit Lomax distribution [21]. By taking *d* = 1, it is a special case of the unit log-logistic distribution [22]. Those models recently appeared in the literature, and the unit Lomax has not been studied in a regression context.

Our proposal is based on new reparametrization on *Y* by inverting its quantile function. We provide at least four motivations for this work. First, we propose a new reparametrization on *Y* and derive some useful statistical quantities that were not explored by [8]. Our investigation includes the computation of the score function and observed information matrix for distribution and also for the regression. Second, we consider a regression structure for the new quantile parameterby assuming that it can be expressed as a function of covariates and, hence, a more general class of regressions is obtained. The third motivation is to use a statistical learning tool for comparing the prediction performance of non-nested models and selecting the most suitable for the data at hand. The fourth motivation is referring to the usefulness of the new regression for modeling the dropout proportion of undergraduate courses. The motivating data set concern to Brazilian undergraduate animal science courses. This course has received attention in the literature; see, for instance, [23], who sought to identify demographic variables as well as their relation to students’ performance and interest areas, and factors associated with enrollment in an introductory animal sciences course.

The rest of paper is outlined as follows. Section 2 proposes an alternative quantile parameterization for the UBXII distribution and investigates some of its mathematical and statistical properties. We obtain the maximum likelihood of the parameters in Section 3. We provide a simulation study in Section 4 to evaluate the performance of the estimators. In Section 5, we define a quantile regression model based on the new parameterization of the UBXII distribution. In addition, we discuss the estimation of the parameters, present some diagnostic analysis methods and regression selection criteria, and conduct simulation studies. In special, we present a statistical learning tool (cross-validation approach) to compare non-nested regressions. In Section 6, we perform an application of the new regression to dropout in Brazilian undergraduate animal sciences courses. We offer some conclusions in Section 7. Finally, we provide the observed matrix for the new distribution and Fisher’s observed information matrix, and information about data’s extraction used in application; see S1 Appendix and Supporting Information, respectively.

## 2 A new UBXII parametrization

Distributions with direct interpretation parameters are desirable in empirical applications, and for this purpose, several authors have adopted reparameterizations on well-known distributions; see [4, 7, 24, 25]. These reparameterizations generally seek to allow modeling of the random variable’s mean, as in the [4]; and [25] proposal. However, the mean is an outlier-sensitive measure, and the UBXII distribution does not have a closed-form expression for it. Thus, modeling the quantiles is an interesting approach for asymmetric data because they can be outlier-resistant measures [24], besides being a smart alternative since the qf of *Y* (3) has a closed-form, and any quantile can be computed in explicit form. Further, one of the parameters of the UBXII distribution (under a quantile-parameterization) can be interpreted as the *τ*th quantile of *Y*. Thus, we shall reparameterize Eq (1) in terms of the *τ*th quantile *q* = *Q*_*Y*_(*τ*). By inverting (3) and solving for *d*, we have

$$\begin{array}{c} d=\;\text{log}\;\tau^{-1}/\;\text{log}\;\left(1+{\text{log}}^{c}q^{-1}\right). \end{array}$$ (4)

By replacing (4) in Eqs (1) and (2), the cdf and pdf of the UBXII distribution (under this parametrization) have the forms

$$\begin{array}{c} F_{Y}(y;q,c)={\left(1+{\text{log}}^{c}y^{-1}\right)}^{\;\text{log}\;\tau /\;\text{log}\;(1+{\text{log}}^{c}\;q^{-1})},\;0<y<1, \end{array}$$ (5)

and

$$\begin{array}{c} f_{Y}\left(y;q,c\right)=\frac{\;\text{log}\;\tau^{-c}{\text{log}}^{c-1}y^{-1}}{y\;\text{log}\;(1+{\text{log}}^{c}q^{-1})}{\left(1+{\text{log}}^{c}y^{-1}\right)}^{\;\text{log}\;\tau /\;\text{log}\;(1+{\text{log}}^{c}\;q^{-1})-1}, \end{array}$$ (6)

respectively. Henceforth, we denote a random variable with density (6) by *Y*∼ UBXII (*c*, *q*).

**Fig 1:**
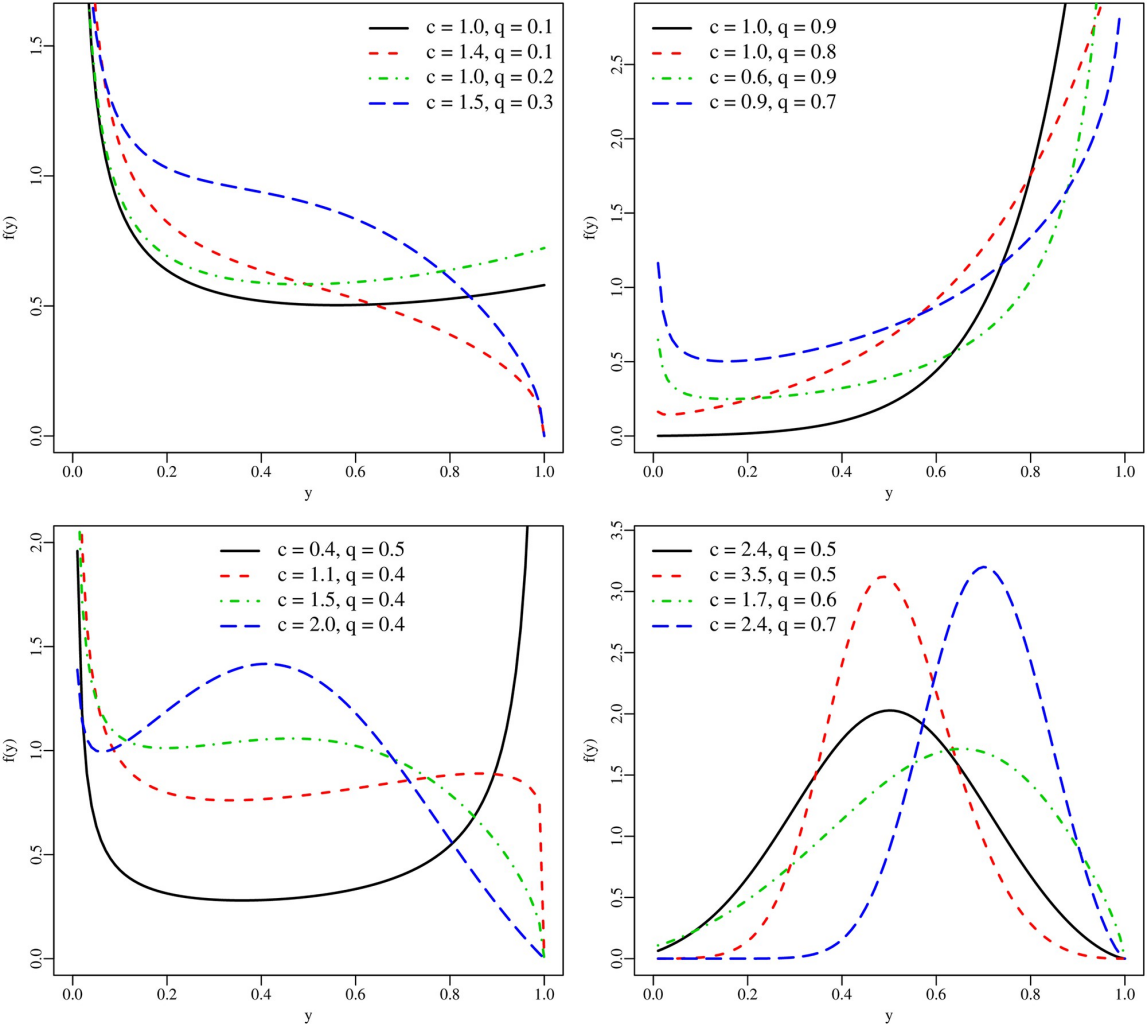
Plots of the UBXII density (with *τ* = 0.5).

Some UBXII densities (for *τ* = 0.5) are displayed in Fig 1, which reveal different shapes such as decreasing, increasing, reverse J-shaped, U-shaped, reverse tilde-shaped (decreasing-increasing-decreasing), non-skewed, and skewed-left. It is noteworthy that the UBXII density can accommodate several skew-left shapes and has a reverse tilde-shaped, which is not presented by classical unit distributions.

The qf of *Y* on the new parameterization has the form

$$\begin{array}{c} Q_{Y}(u)=\text{exp}\{-{\left[u^{\;\text{log}\;(1+{\text{log}}^{c}q^{-1})/\;\text{log}\;\tau }-1\right]}^{1/c}\},\;0<\tau <1. \end{array}$$ (7)

So, the UBXII quantiles can be obtained from (7) by setting *u* values. Further, we can generate occurrences for this distribution using (7) by the inversion method.

**Fig 2:**
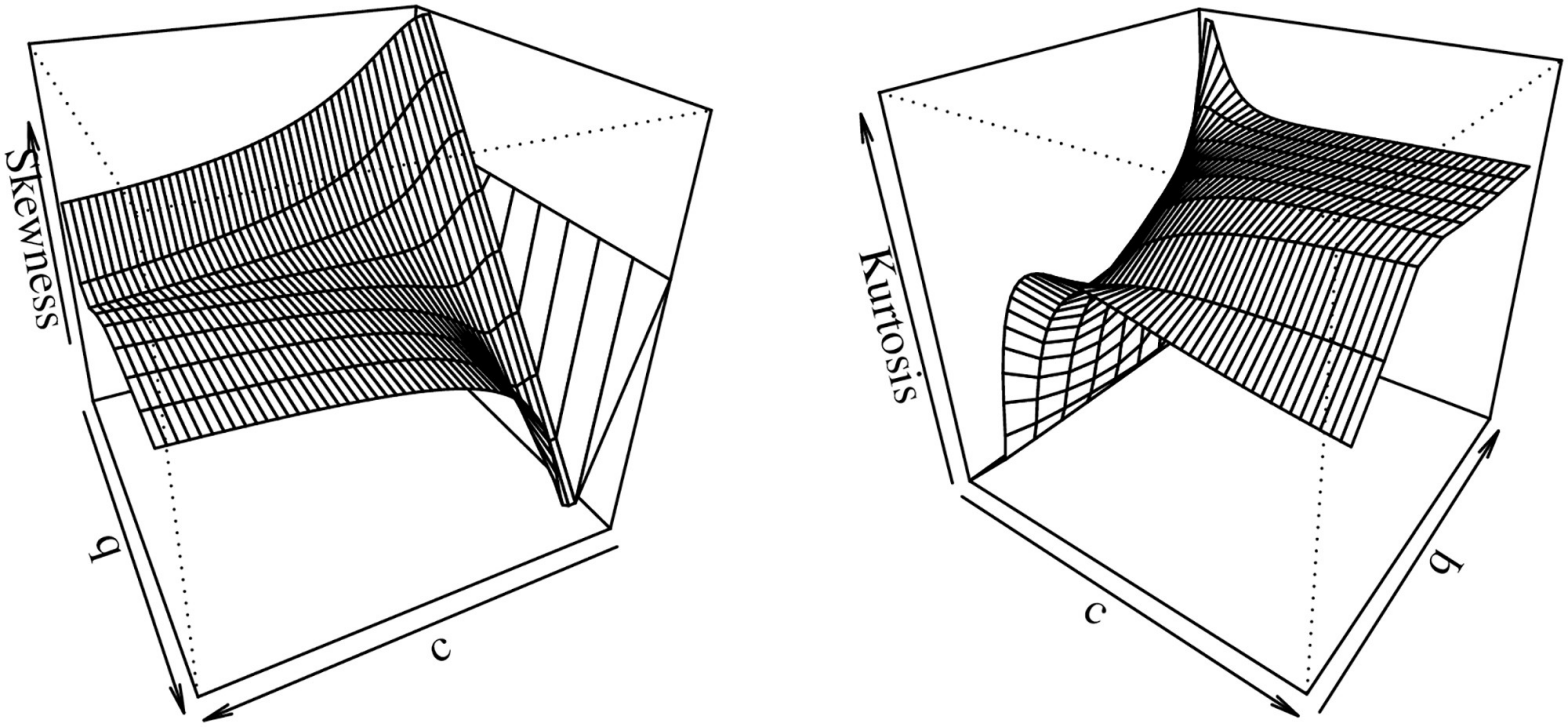
The Bowley skewness and Moors kurtosis of the UBXII distribution.

Alternatively, the flexibility of the new distribution can be displayed from the Bowley skewness and Moors kurtosis formulas, namely

$$B=\frac{Q_{Y}\left(3/4\right)-2Q_{Y}\left(1/2\right)+Q_{Y}\left(1/4\right)}{Q_{Y}\left(3/4\right)-Q_{Y}\left(1/4\right)}$$

and

$$M=\frac{Q_{Y}\left(7/8\right)-Q_{Y}\left(5/8\right)+Q_{Y}\left(3/8\right)-Q_{Y}\left(1/8\right)}{Q_{Y}\left(3/4\right)-Q_{y}\left(1/4\right)},$$

respectively, where *Q*_*Y*_(⋅) is the qf given by (7). These measures provide a simple way to figure out the skewness and tail shapes of the distribution. Fig 2 displays plots for both measures *B* and *M* which show that they are sensible to variations of *c* and *q* for fixed *τ* = 0.5.

## 3 Estimation

Various methods can be used to estimate the parameters of a distribution. The maximum likelihood (ML) method is the most commonly used. In what follows, we shall use this method for estimating the parameters of the UBXII distribution.

Let *y*_1_, …, *y*_*n*_ be a random sample of size *n* from the UBXII distribution, the parameter vector ***θ*** = (*c*, *q*)^⊤^, and a known *τ* ∈ (0, 1) specified. Based on this sample, the log-likelihood function for ***θ***, *ℓ*(***θ***;*y*) ≡ *ℓ*(***θ***), has the form

$$\begin{array}{cc} \ell (\theta )=\; & n\;\;\text{log}\;\left(\;\text{log}\;\tau^{-c}\right)-n\;\text{log}\;\left\{\;\text{log}\;\left[t\left(q\right)\right]\right\}-\sum_{i=1}^{n}\;\;\text{log}\;y_{i}+\left(c-1\right)\sum_{i=1}^{n}\;\;\text{log}\;\left(\;\text{log}\;y_{i}^{-1}\right)\\ & -[1+\frac{\;\text{log}\;\tau^{-1}}{\;\text{log}\;[t(q)]}]\sum_{i=1}^{n}\;\text{log}\;\left[t\left(y_{i}\right)\right], \end{array}$$ (8)

where *t*(*x*) = 1 + log^*c*^ *x*^−1^.

Eq (8) can be maximized either directly by using well-known plataforms such as the R (optim function), SAS (PROC NLMIXED), Ox program (MaxBFGS sub-routine) or by solving the nonlinear likelihood equations from the differentiation of *ℓ*(***θ***). By maximizing (8), we obtain the MLE $\hat{\theta }$ of ***θ***.

**Fig 3:**
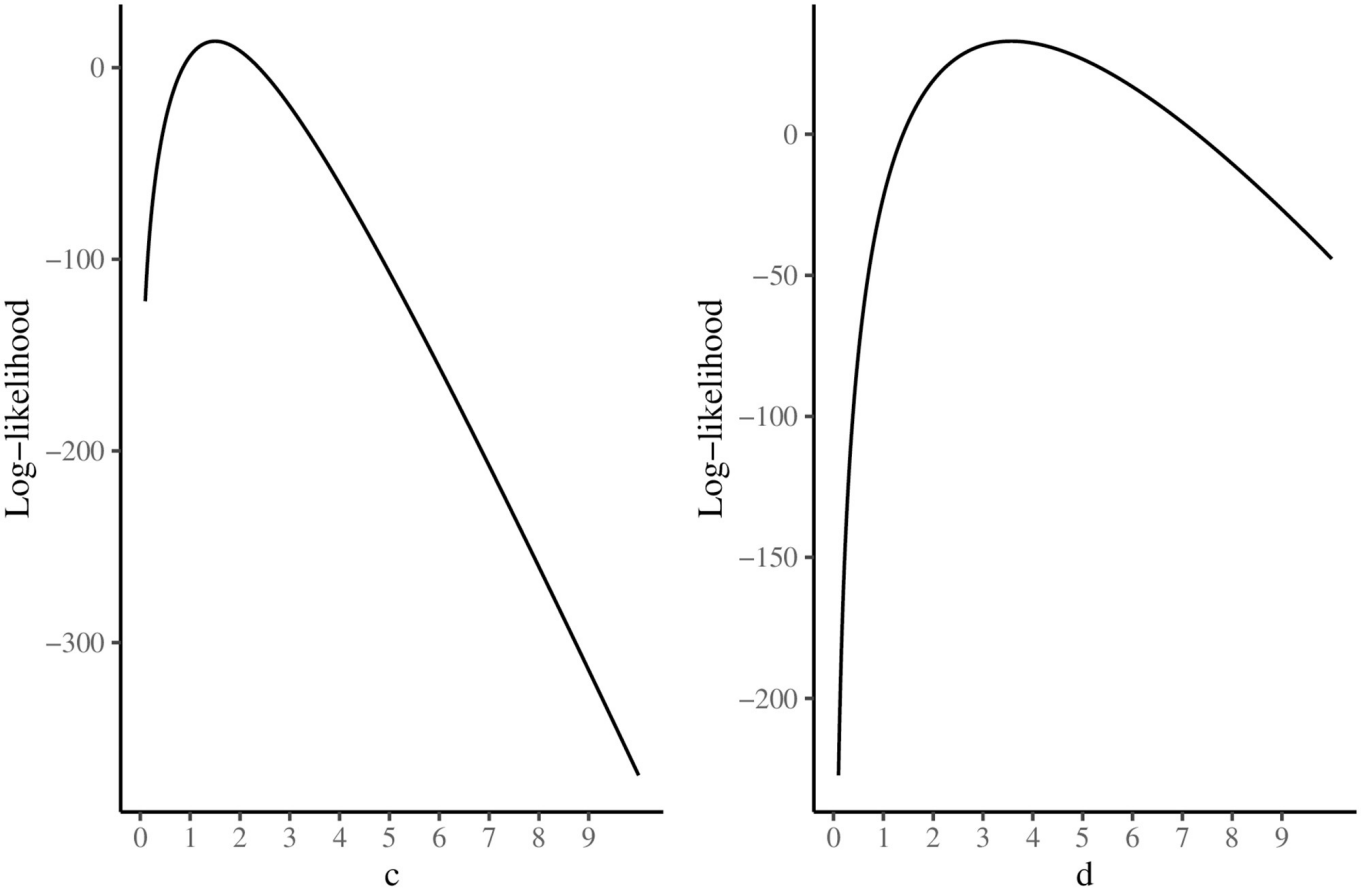
Plots of the log-likelihood function against the parameters *c* and *d*.

Graphically, it is possible to show local maxima of the log-likelihood function ($\hat{\theta }$) and that it is unimodal. Plots that illustrate this are constructed in four steps. First, we simulate data from UBXII(*c*, *d*), where *c* = 1.5 and *d* = 3.4 with *n* = 100. Second, we evaluate the log-likelihood function obtained from the pdf of Eq (2) in a range covering the respective ML estimate, that is, *c* ∈ (0, 9) for *d* fixed at 3.4. After, the same is done for *d* ∈ (0, 9) by fixing *c* at 1.5. Finally, we plot the log-likelihood function against the values range of the parameters *c* and *d*. Fig 3 displays the plots obtained. As expected, both log-likelihood functions are unimodal and their maxima points (ML estimates) are achieved on the true values of *c* and *d*, respectively.

The components of the score vector from Eq (8) are *U*(***θ***) = [*U*_*c*_(***θ***), *U*_*q*_(***θ***)]^⊤^, where *U*_*c*_(***θ***) = ∂*ℓ*(***θ***)/∂*c* and *U*_*q*_(***θ***) = ∂*ℓ*(***θ***)/∂*q*. Setting these components to zero and solving them simultaneously gives $\hat{\theta }$. The score components are

$$\begin{array}{cc} U_{c}\left(\theta \right)= & \;\frac{n}{c}+\sum_{i=1}^{n}\;\;\text{log}\;\left(\;\text{log}\;y_{i}^{-1}\right)-\frac{n\;\;\text{log}\;\left(\;\text{log}\;q^{-1}\right)\left[t\left(q\right)-1\right]}{t(q)\;\text{log}\;[t(q)]}-\sum_{i=1}^{n}\frac{\left[t\left(y_{i}\right)-1\right]\;\text{log}\;\left(\;\text{log}\;y_{i}^{-1}\right)}{t(y_{i})}\\ & -\frac{\;\text{log}\;\tau^{-1}\;\text{log}\;\left[t\left(q\right)\right]}{{\text{log}}^{2}\left[t\left(q\right)\right]}\sum_{i=1}^{n}{\left[t\left(y_{i}\right)\right]}^{-1}\left[t\left(y_{i}\right)-1\right]\;\text{log}\;\left(\;\text{log}\;y_{i}^{-1}\right)\\ & +\frac{\;\text{log}\;\tau^{-1}\left[t\left(q\right)-1\right]\;\text{log}\;\left(\;\text{log}\;q^{-1}\right)}{t\left(q\right){\text{log}}^{2}\left[t\left(q\right)\right]}\sum_{i=1}^{n}\;\text{log}\;\left[t\left(y_{i}\right)\right], \end{array}$$

and

$$\begin{array}{c} U_{q}\left(\theta \right)=\;\frac{n\;c{\text{log}}^{c-1}q^{-1}}{q\;t(q)\;\text{log}\;[t(q)]}-\frac{\;\text{log}\;\tau^{-c}{\text{log}}^{c-1}q^{-1}}{q\;t\left(q\right){\text{log}}^{2}\left[t\left(q\right)\right]}\sum_{i=1}^{n}\;\text{log}\;\left[t\left(y_{i}\right)\right]. \end{array}$$

The MLE of ***θ*** can not be expressed in closed-form by setting ${U\left(\theta \right)|}_{\theta =\hat{\theta }}=0$. However, for fixed *c*, we note that a MLE semi-closed form of *q* follows by taking $U_{q}{\left(\theta \right)|}_{q=\hat{q}}=0$. Hence, it is the solution of

$$\begin{array}{c} \hat{q}\left(c\right)=\text{exp}(-{\left\{\text{exp}[\frac{1}{n}\;\text{log}\;\tau^{-1}\sum_{i=1}^{n}\;\text{log}\;\left[t\left(y_{i}\right)\right]]-1\right\}}^{1/c}). \end{array}$$

By replacing *q* by $\hat{q}\left(c\right)$ in Eq (8), we obtain the profile log-likelihood function

$$\begin{array}{cc} \ell (c)=-n+ & n\;\;\text{log}\;\left(\;\text{log}\;\tau^{-c}\right)-\sum_{i=1}^{n}\;\;\text{log}\;y_{i}-\sum_{i=1}^{n}\;\text{log}\;\left[t\left(y_{i}\right)\right]+\left(c-1\right)\sum_{i=1}^{n}\;\;\text{log}\;\left(\;\text{log}\;y_{i}^{-1}\right)\\ & -n\;\text{log}\;\;\{\frac{1}{n}\;\text{log}\;\tau^{-1}\sum_{i=1}^{n}\;\text{log}\;\left[t\left(y_{i}\right)\right]\}. \end{array}$$ (9)

We can compute the score function for *c* from (9)

$$\begin{array}{c} U_{c}\left(c\right)=\frac{n}{c}+\sum_{i=1}^{n}\;\text{log}\;\left(\;\text{log}\;y_{i}^{-1}\right)-\sum_{i=1}^{n}\frac{\left[t\left(y_{i}\right)-1\right]\;\text{log}\;\left(\;\text{log}\;y_{i}^{-1}\right)}{t(y_{i})}-\frac{n\sum_{i=1}^{n}\frac{\left[t\left(y_{i}\right)-1\right]\;\text{log}\;\left(\;\text{log}\;y_{i}^{-1}\right)}{t(y_{i})}}{\sum_{i=1}^{n}\;\text{log}\;\left[t\left(y_{i}\right)\right]}. \end{array}$$

However, it is necessary to use a nonlinear optimization method to maximize numerically the profile log-likelihood function (9). Typically for the numerical computation of the MLEs, the quasi-Newton algorithm such as Broyden-Fletcher-Goldfarb-Shanno (BFGS) algorithm is adopted.

Approximate confidence intervals and hypothesis tests for ***θ*** can be constructed by considering its asymptotic distribution of the MLEs. For large samples, $\hat{\theta }∼N\left(0,I^{-1}\left(\theta \right)\right)$ approximately assuming that standard regularity conditions (SRCs) hold, where *I*(***θ***) is the expected information matrix defined by

$$I\left(\theta \right)=I\;E(-\frac{\partial \ell (\theta )}{\partial \theta }\frac{\partial \ell (\theta )}{\partial \theta^{⊤}}).$$

The computation of *I*(***θ***) may be cumbersome. Nevertheless, when the SRCs are valid, it follows that I(θ)=IE[*J*(***θ***)], where *J*(***θ***) = −∂^2^ *ℓ*(***θ***)/∂***θ***∂***θ***^⊤^ is the observed information matrix. For the UBXII distribution, we can write *J*(***θ***) as

$$J\left(\theta \right)=-[\begin{array}{cc} U_{cc}\left(\theta \right) & \;\;U_{cq}\left(\theta \right)\\ U_{qc}\left(\theta \right) & \;\;U_{qq}\left(\theta \right) \end{array}],$$

where *U*_*cc*_(***θ***) = ∂^2^ *ℓ*(***θ***)/∂*c*^2^, *U*_*qq*_(***θ***) = ∂^2^ *ℓ*(***θ***)/∂*q*^2^, and *U*_*cq*_(***θ***) = ∂^2^ *ℓ*(***θ***)/(∂*c*∂*q*) = *U*_*qc*_(***θ***). The elements of the matrix *J*(***θ***) are given in S1 Appendix.

[26] proved that the estimated observed information matrix $J(\hat{\theta })$ is a consistent estimator of *I*(***θ***) when the sample size is large. It is then possible to obtain the standard errors (SEs) of the MLEs by computing the square roots of the diagonal elements of $J{\left(\hat{\theta }\right)}^{-1}$. For instance, we can do large sample inference by building asymptotic confidence intervals with 100%(1 − *α*) nominal coverage for ***θ*** making $\hat{\theta }\pm z_{1-\alpha /2}SE\left(\hat{\theta }\right)$, where *z*_1−*α*/2_ is the 1 − *α*/2 standard normal quantile.

## 4 Simulation study

A Monte Carlo simulation study is carried out in the R programming language to evaluate the performance of the MLEs of the UBXII parameters that index the distribution. The Optim routine (with BFGS quasi-Newton nonlinear optimization algorithm and analytical derivative) is used for maximizing (9). The profile log-likelihood function involves a more straightforward numerical maximization than using (8) since it depends only on the parameter *c*. We start the root-finding algorithm using *c* = 1 for the shape parameter.

Different values for the parameter vector ***θ*** are considered according to those presented in Fig 1. Therefore, various combinations of skewness and kurtosis coefficients and density shapes are contemplated. A total of six scenarios is considered for the sample size *n* ∈ {25, 75, 150, 300}. The inversion method is employed for generating observations, i.e., the qf (7) is evaluated in u∼U(0,1), being *Q*_*Y*_(*u*) = *y* and, hence, a sample of size *n* from *Y*∼ UBXII (*c*, *q*) is generated. Each one of the sample sizes is replicated *R* = 10, 000 times. We compute quantities as percentage relative bias (RB%) and root mean squared error (RMSE) of the MLEs.

Table 1 reports results from the simulation schemes. As expected, the consistency property of the MLEs holds, i.e., the RMSEs tend to decrease when the sample size increases. Also, it can be noted that the RB%s are smaller for sample size higher, thus indicating that the overall performance of the MLEs is appropriate, as well as they are more accurate and less biased when *n* increases. Notice that the biggest RB%s for $\hat{c}$ and $\hat{q}$ are less than 7.38 and 1.62, respectively, even with *n* = 25. In general, the estimate $\hat{q}$ is more accurate when compared with $\hat{c}$. In the scenarios two to six, all the RB%s of $\hat{q}$ are below of 0.84 in absolute value.

**Table 1:** RB%s and RMSEs from the UBXII distribution.

Scenario	*c*	*q*	*n*	RB%		RMSE	
				$\hat{c}$	$\hat{q}\left(\hat{c}\right)$	$\hat{c}$	$\hat{q}\left(\hat{c}\right)$
1	1.5	0.3	25	7.3773	1.6170	0.3682	0.0751
			75	2.3671	0.8954	0.1823	0.0440
			150	1.1971	0.5372	0.1251	0.0310
			300	0.6399	0.3746	0.0872	0.0225
2	0.9	0.7	25	5.1296	−0.8436	0.1598	0.0758
			75	1.6937	−0.2415	0.0845	0.0434
			150	0.7708	−0.1126	0.0585	0.0311
			300	0.4013	−0.0741	0.0409	0.0221
3	1.1	0.4	25	6.3920	0.6669	0.2370	0.0967
			75	2.1153	0.6017	0.1216	0.0569
			150	1.1037	0.3580	0.0833	0.0402
			300	0.6429	0.3446	0.0583	0.0290
4	2.0	0.4	25	6.0735	0.2902	0.4203	0.0541
			75	1.9732	0.1424	0.2169	0.0313
			150	0.8997	0.0865	0.1496	0.0224
			300	0.4774	0.0212	0.1049	0.0159
5	1.7	0.6	25	5.0479	−0.1864	0.2940	0.0481
			75	1.6641	−0.0366	0.1555	0.0276
			150	0.7552	−0.0085	0.1075	0.0198
			300	0.3920	−0.0157	0.0755	0.0140
6	3.5	0.5	25	5.0270	0.0122	0.5975	0.0262
			75	1.6559	0.0203	0.3157	0.0150
			150	0.7520	0.0171	0.2182	0.0108
			300	0.3894	0.0012	0.1532	0.0076

**Fig 4:**
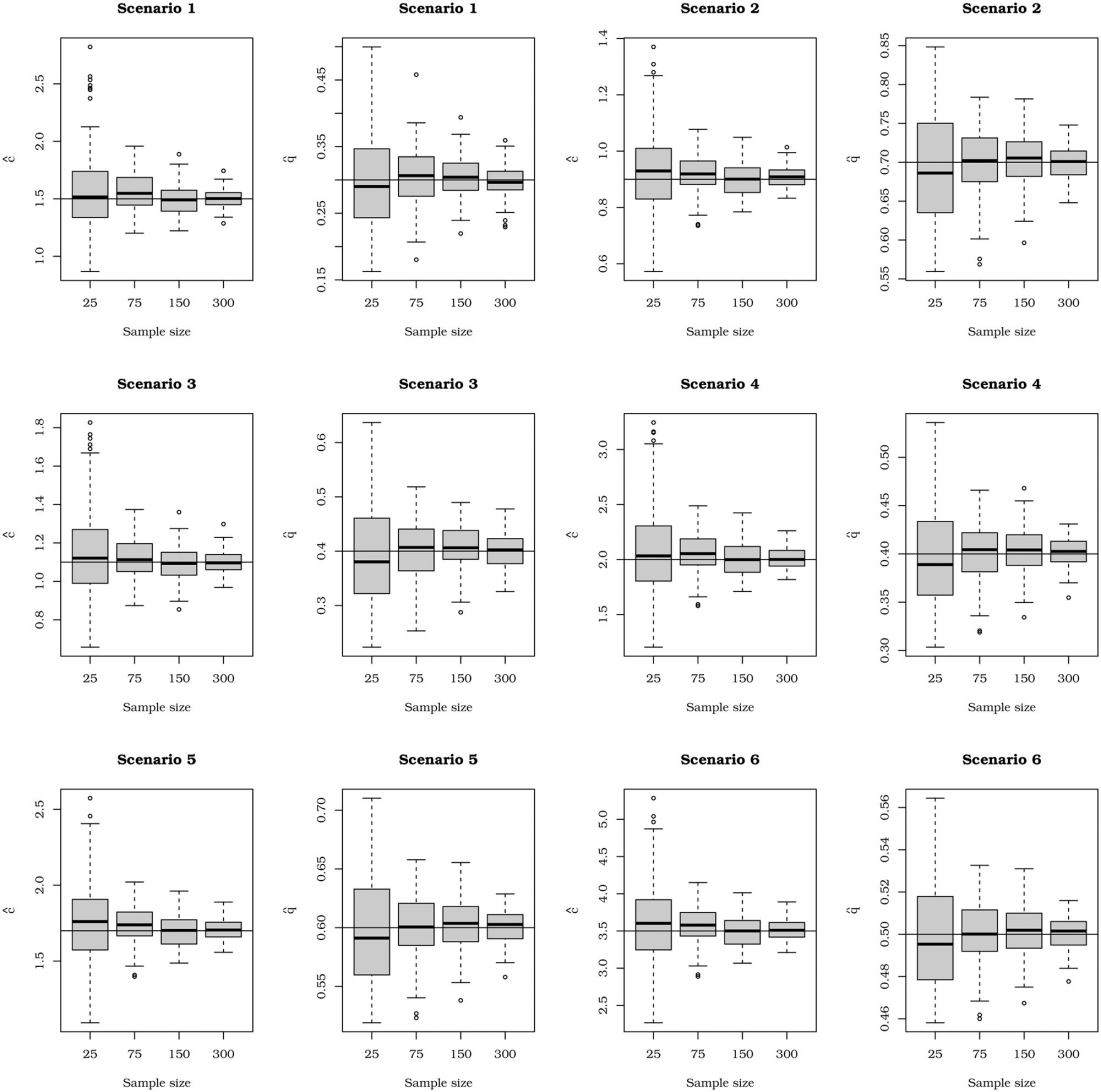
Boxplots of the first hundred estimates of the Monte Carlo simulation for some sample sizes.

Fig 4 displays boxplots from the first 100 Monte Carlo replications (to favor easy viewing) of the eight current scenarios. We can note that, in most cases, the presence of outliers overestimates the estimates for small sample sizes. However, this fact is attenuated when *n* increases. Besides, the dispersion of the estimates decreases, and the precision is achieved for larger sample sizes.

**Fig 5:**
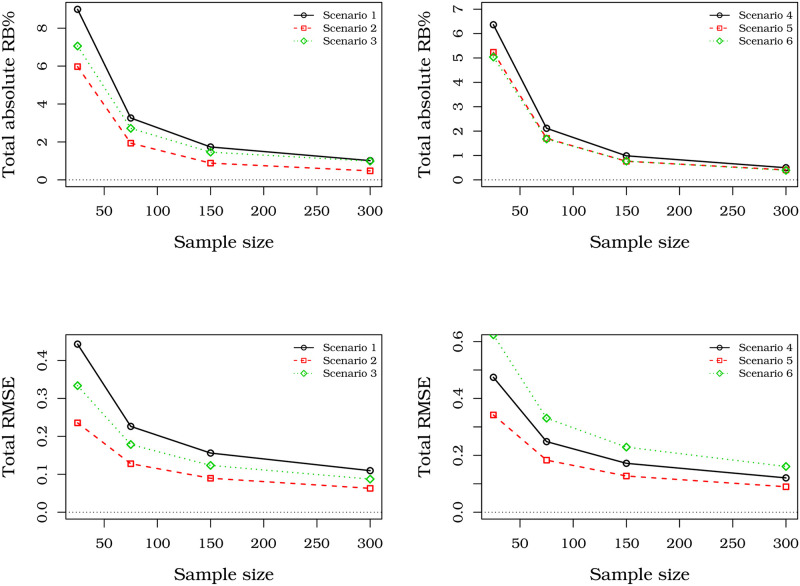
Total absolute RB%s and total RMSE of the MLEs from UBXII distribution with different sample sizes.

Fig 5 contains plots of total absolute RB% and total RMSE versus sample sizes for all these scenarios. These quantities are obtained from the sum of the RB% and RMSE of both parameters for each sample size and scenario. Note that those measures decay to zero when *n* increases in the six scenarios. This shows that the properties of the MLEs (such as asymptotically unbiased and consistent) are held.

## 5 The UBXII regression

Let *Y*_1_, …, *Y*_*n*_ be *n* independent random variables, where *Y*_*i*_∼ UBXII (*q*_*i*_, *c*) for *i* = 1, …, *n* with shape parameter *c* and quantile parameter *q*_*i*_ (both unknown) for 0 < *τ* < 1 assumed known. We propose the *UBXII regression* imposing that the quantile *q*_*i*_ of *Y*_*i*_ satisfies the functional relation

$$\begin{array}{c} \eta =g(q)=X\beta , \end{array}$$ (10)

where $\eta ={\left(\eta_{1},\ldots ,\eta_{n}\right)}^{⊤}\in {I\;R}^{n}$ is the *n*-dimensional vector of linear predictors, *q* = (*q*_1_, …, *q*_*n*_)^⊤^ is the vector of quantiles with *q*_*i*_ ∈ (0, 1), $\beta ={\left(\beta_{1},\ldots ,\beta_{k}\right)}^{⊤}\in {I\;R}^{k}$ is a *k*-dimensional vector of unknown regression coefficients (*k* < *n*), $X={\left(x_{1}^{⊤},\ldots ,x_{n}^{⊤}\right)}^{⊤}$ is the *n*×*k* full column rank matrix, $x_{i}^{⊤}=\left(x_{i1},\ldots ,x_{ik}\right)$ denotes the *i*th observation on *k* covariates which are assumed known, and *x*_*i*1_ = 1, ∀*i*. Finally, we shall assume that *g*(⋅) is a strictly monotonic and twice differentiable link function which maps (0, 1) into IR. By inverting each component of (10), we can write

$$q_{i}=g^{-1}(\sum_{j=1}^{k}x_{ij}\beta_{j})=g^{-1}\left(\eta_{i}\right).$$

There are various possible choices for the link function *g*(⋅) such as

- logit: *g*(*q*_*i*_) = log[*q*_*i*_/(1−*q*_*i*_)];
- probit: *g*(*q*_*i*_) = Φ^−1^(*q*_*i*_), where Φ^−1^(⋅) is the qf of the standard normal random variable;
- complementary log-log: *g*(*q*_*i*_) = log[−log(1−*q*_*i*_)];
- log-log: *g*(*q*_*i*_) = −log[−log(*q*_*i*_)];
- Cauchy: *g*(*q*_*i*_) = tan[*π*(*q*_*t*_−1/2)].

The choice of the logit link function is the most common by practitioners since the interpretation of the regression parameters becomes quite interesting. Consider increasing the *j*th regressor at one unit, while the others are kept constant. Let q* be the quantile of *Y* under the new value of *x*_*j*_, whereas *q* denotes the quantile of *Y* under the original value of this regressor. It can be shown that with the logit link function, we have *β*_*j*_ = log{q*(1−q*)/[*q*(1−*q*)]}, i.e., *β*_*j*_ is the log odds ratio [4]. In this context, we will consider the logit link function for *g*(⋅) in the UBXII regression. Then, the *i*th quantile of *Y*_*i*_ is $q_{i}=e^{\eta_{i}}/(1+e^{\eta_{i}})$.

### 5.1 Estimation

The parameters estimation in the UBXII regression can also be performed by the ML method. Let ***θ*** = (***β***^⊤^, *c*)^⊤^ be the vector of *k* + 1 unknown parameters to be estimated. The log-likelihood function based on a sample of *n* independent observations having the UBXII distribution, i.e., *Y*_*i*_∼ UBXII (*q*_*i*_, *c*), can be expressed as

$$\begin{array}{c} \ell \left(\theta \right)\equiv \ell \left(\beta ,c\right)=\sum_{i=1}^{n}\ell_{i}\left(q_{i},c\right), \end{array}$$ (11)

where *ℓ*_*i*_(*q*_*i*_, *c*) is the logarithm of *f*_*Y*_(*y*_*i*_;*q*_*i*_, *c*) given in Eq (6). Hence,

$$\begin{array}{cc} \ell_{i}\left(q_{i},c\right)= & \;\text{log}\;\left(\;\text{log}\;\tau^{-c}\right)-\;\text{log}\;y_{i}+\left(c-1\right)\;\text{log}\;\left(\;\text{log}\;y_{i}^{-1}\right)-\;\text{log}\;\left[t\left(y_{i}\right)\right]-\;\text{log}\;\left\{\;\text{log}\;\left[t\left(q_{i}\right)\right]\right\}\\ & -\frac{\;\text{log}\;\tau^{-1}\;\text{log}\;\left[t\left(y_{i}\right)\right]}{\;\text{log}\;[t\left(q_{i}\right)]}. \end{array}$$

The score vector, obtained by differentiating the log-likelihood function (11) with respect to the unknown parameters *β*_*j*_, *j* = 1, …, *k*, and *c*, is expressed as ***U*** = [*U*_*β*_(***β***, *c*)^⊤^, *U*_*c*_(***β***, *c*)]^⊤^. The components of ***U*** can be written in matrix notation. For doing this, we now define some quantities.

Let $q_{i}^{⋆}={\text{log}}^{c-1}q_{i}^{-1}/\left\{q_{i}\;t\left(q_{i}\right)\;\text{log}\;\left[t\left(q_{i}\right)\right]\right\}$, $q_{i}^{†}=\;\text{log}\;\tau^{-1}{\text{log}}^{c-1}q_{i}^{-1}/\left\{q_{i}\;t\left(q_{i}\right){\text{log}}^{2}\left[t\left(q_{i}\right)\right]\right\}$, $y_{i}^{⋆}=\;\text{log}\;\left[t\left(y_{i}\right)\right]$, and

$$\begin{array}{cc} y_{i}^{♯}= & \;\frac{1}{c}+\;\text{log}\;\left(\;\text{log}\;y_{i}^{-1}\right)-\frac{\;\text{log}\;\left(\;\text{log}\;q_{i}^{-1}\right)\left[t\left(q_{i}\right)-1\right]}{t\left(q_{i}\right)\;\text{log}\;\left[t\left(q_{i}\right)\right]}-\frac{\left[t\left(y_{i}\right)-1\right]\;\text{log}\;\left(\;\text{log}\;y_{i}^{-1}\right)}{t(y_{i})}\\ & -\frac{\;\text{log}\;\tau^{-1}\;\text{log}\;\left[t\left(q_{i}\right)\right]{\left[t\left(y_{i}\right)\right]}^{-1}\left[t\left(y_{i}\right)-1\right]\;\text{log}\;\left(\;\text{log}\;y_{i}^{-1}\right)}{{\text{log}}^{2}\left[t\left(q_{i}\right)\right]}\\ & +\frac{\;\text{log}\;\tau^{-1}\left[t\left(q_{i}\right)-1\right]\;\text{log}\;\left(\;\text{log}\;q_{i}^{-1}\right)\;\text{log}\;\left[t\left(y_{i}\right)\right]}{t\left(q_{i}\right){\text{log}}^{2}\left[t\left(q_{i}\right)\right]}. \end{array}$$

Then, we have

$$\begin{array}{c} U_{\beta }\equiv U_{\beta }\left(\beta ,c\right)=c\;X^{⊤}\;D\;\left(\;q^{⋆}-q^{†}\;y^{⋆}\right), \end{array}$$ (12)

and

$$\begin{array}{c} U_{c}\equiv U_{c}\left(\beta ,c\right)=\text{tr}\left(Y^{♯}\right), \end{array}$$ (13)

where ***X*** is an *n* × *k* matrix whose *i*th row is $x_{i}^{⊤}$, ***D*** = diag {1/*g*′(*q*_1_), …, 1/*g*′(*q*_*n*_)}, $q^{⋆}={\left(q_{1}^{⋆},\ldots ,q_{n}^{⋆}\right)}^{⊤}$, $q^{†}={\left(q_{1}^{†},\ldots ,q_{1}^{†}\right)}^{⊤}$, $y^{⋆}={\left(y_{1}^{⋆},\ldots ,y_{n}^{⋆}\right)}^{⊤}$, and $Y^{♯}=\text{diag}\left\{y_{1}^{♯},\ldots ,y_{n}^{♯}\right\}$. We provide the calculations of the score components in S1 Appendix.

Again, the nonlinear Equations $U_{\beta }{|}_{\beta =\hat{\beta }}=0$ and $U_{c}{|}_{c=\hat{c}}=0$ can not be expressed in closed-form. Hence, a nonlinear optimization method must be used for maximizing the function (11) and determine the MLEs ${\left({\hat{\beta }}^{⊤},\hat{c}\right)}^{⊤}$. We also provide the observed information matrix for (***β***^⊤^, *c*)^⊤^.

To simplify the notation of its components, other quantities are defined as follows

$$\begin{array}{c} m_{i}=\{\frac{c\;{\text{log}}^{c}q_{i}^{-1}}{q_{i}\;t\left(q_{i}\right)}+\frac{c{\text{log}}^{c}q_{i}^{-1}}{q_{i}\;t\left(q_{i}\right)\;\text{log}\;\left[t\left(q_{i}\right)\right]}-\frac{\;\text{log}\;q_{i}^{-1}}{q_{i}}-\frac{\left(c-1\right)}{q_{i}}\}\frac{c{\text{log}}^{c-2}q_{i}^{-1}}{q_{i}\;t\left(q_{i}\right)\;\text{log}\;\left[t\left(q_{i}\right)\right]}, \end{array}$$

$$\begin{array}{c} p_{i}\;=\;\{\frac{\left(c-1\right)}{q_{i}\;\;\text{log}\;\left[t\left(q_{i}\right)\right]}+\frac{\;\text{log}\;q_{i}^{-1}}{q_{i}\;\;\text{log}\;\left[t\left(q_{i}\right)\right]}-\frac{2\;c{\text{log}}^{c}q_{i}^{-1}}{q_{i}\;t\left(q_{i}\right){\text{log}}^{2}\left[t\left(q_{i}\right)\right]}-\frac{c{\text{log}}^{c}q_{i}^{-1}}{q_{i}\;t\left(q_{i}\right)\;\text{log}\;\left[t\left(q_{i}\right)\right]}\}\;\frac{c\;\text{log}\;\tau^{-1}{\text{log}}^{c-2}q_{i}^{-1}}{q_{i}\;t\left(q_{i}\right)\;\text{log}\;\left[t\left(q_{i}\right)\right]}, \end{array}$$

$$\begin{array}{cc} r_{i}= & \;\{{\text{log}}^{c-1}q_{i}^{-1}+\frac{{\text{log}}^{c-1}q_{i}^{-1}}{c\;\text{log}\;(\;\text{log}\;q_{i}^{-1})}-\frac{{\text{log}}^{2c-1}q_{i}^{-1}}{t(q_{i})}-\frac{{\text{log}}^{2c-1}q_{i}^{-1}}{t\left(q_{i}\right)\;\text{log}\;\left[t\left(q_{i}\right)\right]}\}\frac{c\;\text{log}\;(\;\text{log}\;q_{i}^{-1})}{q_{i}\;t\left(q_{i}\right)\;\text{log}\;\left[t\left(q_{i}\right)\right]}, \end{array}$$

$$\begin{array}{cc} u_{i}= & \left\{\frac{2{\text{log}}^{2c-1}q_{i}^{-1}}{t\left(q_{i}\right){\text{log}}^{2}\left[t\left(q_{i}\right)\right]}+\frac{{\text{log}}^{2c-1}q_{i}^{-1}}{t\left(q_{i}\right)\;\text{log}\;\left[t\left(q_{i}\right)\right]}-\frac{{\text{log}}^{c-1}q_{i}^{-1}}{c\;\text{log}\;\left(\;\text{log}\;q_{i}^{-1}\right)\;\text{log}\;\left[t\left(q_{i}\right)\right]}-\frac{{\text{log}}^{c-1}q_{i}^{-1}}{\;\text{log}\;[t\left(q_{i}\right)]}\right\}\\ & \times \frac{c\;\text{log}\;\left(\;\text{log}\;q_{i}^{-1}\right)\;\text{log}\;\tau^{-1}}{q_{i}\;t\left(q_{i}\right)\;\text{log}\;\left[t\left(q_{i}\right)\right]}, \end{array}$$

$$\begin{array}{c} s_{i}=\frac{c\;\text{log}\;\tau^{-1}{\text{log}}^{c-1}q_{i}^{-1}}{q_{i}\;t\left(q_{i}\right){\text{log}}^{2}\left[t\left(q_{i}\right)\right]}\;\text{and}\;y_{i}^{†}=\;\text{log}\;\left(\;\text{log}\;y_{i}^{-1}\right)\left[t\left(y_{i}\right)-1\right]{\left[t\left(y_{i}\right)\right]}^{-1}. \end{array}$$

Therefore, the observed information matrix can be expressed as (see S1 Appendix)

$$J=-(\begin{array}{cc} J_{\beta \beta } & \;\;J_{c\beta }\\ J_{\beta c} & \;\;J_{cc} \end{array}).$$

The quantities ***J***_***ββ***_ ≡ ∂^2^ *ℓ*(***β***, *c*)/∂***β***∂***β***^⊤^ and $J_{\beta \;c}=J_{c\;\beta }^{⊤}\equiv \partial^{2}\ell \left(\beta ,c\right)/\partial c\partial \beta$, and *J*_*cc*_ ≡ ∂^2^ *ℓ*(***β***, *c*)/∂*c*^2^ are

$$\begin{array}{c} J_{\beta \beta }=X^{⊤}\;\left[\;\left(\;M+P\;Y^{⋆}\right)\;D-c\;\left(Q^{⋆}-Q^{†}Y^{⋆}\right)\;T\;D^{⊤}\;D\;\right]\;D\;X, \end{array}$$ (14)

$$\begin{array}{c} J_{c\;\beta }^{⊤}={\left(r-s\;y^{‡}+u\;y^{⋆}\right)}^{⊤}\;D\;X, \end{array}$$ (15)

and

$$\begin{array}{c} J_{cc}=\text{tr}\left(Y^{◇}\right), \end{array}$$ (16)

where ***M*** = diag {*m*_1_, …, *m*_*n*_}, ***P*** = diag {*p*_1_, …, *p*_*n*_}, $Q^{⋆}=\text{diag}\;\left\{q_{1}^{⋆},\ldots ,q_{n}^{⋆}\right\}$, $Q^{†}=\text{diag}\;\{q_{1}^{†},\ldots ,q_{n}^{†}\}$, $Y^{⋆}=\text{diag}\;\left\{y_{1}^{⋆},\ldots ,y_{n}^{⋆}\right\}$, ***T*** = diag {*g*″(*q*_1_), …, *g*′′(*q*_*n*_)}, ***r*** = (*r*_1_, …, *r*_*n*_)^⊤^, ***s*** = (*s*_1_, …, *s*_*n*_)^⊤^, $y^{‡}={\left(y_{1}^{‡},\ldots ,y_{n}^{‡}\right)}^{⊤}$, and ***u*** = (*u*_1_, …, *u*_*n*_)^⊤^.

As mentioned in Section 3, the matrix ***J*** is quite useful for interval estimation and hypothesis testing inference. Assuming that the SRCs hold and the sample size is large,

$$(\begin{array}{c} \hat{\beta }\\ \hat{c} \end{array})∼N_{k+1}((\begin{array}{c} \beta \\ c \end{array}),\;I^{-1}),$$

where ***I***^−1^ is the inverse of I≡IE(J) is the expected information matrix. It can be estimated of the consistent way by $\hat{J}$, which is computed after replacing the unknown parameters (***β***^⊤^, *c*)^⊤^ by the corresponding MLEs.

### 5.2 Diagnostic measures and model selection

In order to check the goodness-of-fit and validate the UBXII regression assumptions, we adopt some well-known diagnostic tools that are now discussed. Initially, we used quantile residuals. These residuals verify if the model assumptions are satisfied and identify when the parameter estimations are considerably affected by the presence of atypical observations in the response. If the model is correctly specified, the quantile residuals are standard normally distributed. For the UBXII regression, they are given by

$$\begin{array}{c} r_{i}=\Phi^{-1}\left[F_{Y}\left(y_{i};\hat{q_{i}},\hat{c}\right)\right], \end{array}$$

where *F*_*Y*_(⋅) is the UBXII cdf given in Eq (5).

An incorrect functional form specification of the regression and the covariates omission can be identified through the RESET test. This test was initially introduced as a general misspecification test for the normal linear regression. Afterward, variants of the RESET test for classes of more general regressions were proposed by [27]. Thus, to determine whether a UBXII regression is misspecified, we propose using a RESET-like misspecification test. Next, we explain how this test can be performed.

The RESET-like test is carried out in two steps. Let $\hat{q}$ be the predicted values vector obtained after fitting a UBXII regression. First, we build testing variables matrix as $T=[{\hat{q}}^{2}\;,{\hat{q}}^{3}]$, where the vectors ${\hat{q}}^{2}$ and ${\hat{q}}^{3}$ are formed by $\hat{q}$ squared and cubed components, respectively. We define the augmented regression

$$\begin{array}{c} g(q)=X\beta +T\delta , \end{array}$$ (17)

where ***T*** is the *n* × 2 matrix of testing variables, and ***δ*** is a 2 × 1 vector of parameters. Second, we estimate Eq (17) and test the null hypothesis $H_{0}:\delta =0$ against the alternative hypothesis $H_{1}:\delta \neq 0$ by using the likelihood ratio (LR) statistic. We compute the LR statistic as $\omega =2[\ell \left(\hat{\theta }-\ell \left(\tilde{\theta }\right)\right],$ where *ℓ*(⋅) is the log-likelihood function and $\hat{\theta }={\left({\hat{\delta }}^{⊤},{\hat{\beta }}^{⊤},\hat{c}\right)}^{⊤}$ is the unrestricted MLE of ***θ***, and $\tilde{\theta }={\left(0^{⊤},{\tilde{\beta }}^{⊤},\tilde{c}\right)}^{⊤}$ is the restricted MLE of ***θ*** under the null hypothesis. Under $H_{0}$ and the SRCs, *ω* converge in distribution to chi-square, $\chi_{\nu }^{2}$, where *ν* is the number of testing covariates added to the regression (*ν* = 2 in this case). The non-rejection of the null hypothesis suggests that the regression is correctly specified.

The proportion of the response variable’s variability explained by a fitted UBXII regression can be assessed using the generalized (pseudo) R-squared ($R_{G}^{2}$) defined by [28] as

$$\begin{array}{c} R_{G}^{2}=1-\text{exp}\{-2/n\;[\ell \left(\hat{\theta }\right)-\ell \left(\hat{\theta_{0}}\right)]\}, \end{array}$$

where $\ell ({\hat{\theta }}_{0})$ is the log-likelihood of the null regression, i.e., obtained from the modeling of the response in the covariates absence, and $\ell (\hat{\theta })$ is the log-likelihood of the full regression. A regression with a higher value of $R_{G}^{2}$ provides a larger explanation power of the response variable variation.

To select the more suitable model between several nested models, the information criteria such as Akaike information criterion (AIC) and Schwarz information criterion (BIC) can be considered. Both criteria are widely used in practical applications and they are defined by AIC $\left(\phi \right)=2\;[\;p-\ell \left(\hat{\theta }\right)]$ and BIC $=p\;\text{log}\;\;n-2\ell (\hat{\theta })$, where *p* is the number of estimated parameters.

A way of selecting the best one between different non-nested regressions is to assess its performance in the prediction of the response through statistical learning tools such as the cross-validation approach. Let *y* = (*y*_1_, …, *y*_*n*_)^⊤^ the vector of *n* observations of a response variable and *X* the covariates matrix like in (10). In statistical learning methods, a training data set is the observations set in which a model is initially adjusted. An accuracy measure is the *test error*, that result from applying the model fitted to test observations that were not used in training. For example, if we use (***y***, ***X***) as training observations, the test error is $I\;E[L\left(Y_{0},{\hat{y}}_{0}\right)]$, where *L*(⋅) is the loss function and ${\hat{y}}_{0}$ is the predicted value using the fitted model from (***y***, ***X***) evaluated in the predictors ${x_{0}}^{⊤}$ (that does not belong to ***X***). To estimate the test error with quadratic loss, we consider the mean square error (MSE) defined as

$$\begin{array}{c} \text{MSE}=\frac{1}{n}\sum_{i=1}^{n}{\left(y_{i}-{\hat{y}}_{i}\right)}^{2}, \end{array}$$

where ${\hat{y}}_{i}$ is the *i*th predict value by the regression for the *i*th observation. This statistical measure is small if the predictions of the responses are very close to its true values, and it is large if for some of the observations, the predicted and true responses differ substantially [29].

As cross-validation method, we propose the use of the leave-one-out cross-validation (LOOCV). In this approach, we split the *i*th observation (*i*th row of a data set in which the response and covariates are disposed by columns) of the other *n*−1 observations that represent the training set whereas the row *i* is the validation set.

For each removed observation, we use the fitted model with the training set to predict the *i*th observation of the validation set. After, we estimate the test error by computing the MSE*i*. Repeating those procedure *n* times, we obtain MSE_1_, …, *MSE*_*n*_. The final estimate of the test errors are computed through average of those *n* statistics as follows [29]

$${\text{CV}}_{\left(n\right)}=\frac{1}{n}\sum_{i=1}^{n}{\text{MSE}}_{i}.$$

Hence, we select the regression which provides smaller values for CV_(*n*)_.

Finally, we perform an influence analysis to detect possible influential points as outliers. For this, the generalized Cook distance (GD) is considered. It is a measure of global influence, which proposes eliminating the *i*th observation (*i* = 1, …, *n*) to study its effect. The GD is computed as

$$\begin{array}{c} GD_{i}={\left({\hat{\theta }}_{\left(i\right)}-\hat{\theta }\right)}^{⊤}\left[J\left(\hat{\theta }\right)\right]\left({\hat{\theta }}_{\left(i\right)}-\hat{\theta }\right), \end{array}$$ (18)

where ${\hat{\theta }}_{\left(i\right)}$ is the MLE obtained when the *i*th observation is deleted, and $J(\hat{\theta })$ is the observed information matrix evaluated on the MLEs. We consider a general rule of thumb as a threshold for determining highly influential points. The rule is the following if *GD*_*i*_ > 4/*n*, then the observation is influential.

### 5.3 Simulation study

In this section, a Monte Carlo simulation study is conducted in order to numerically evaluate the finite sample behavior of the MLEs of the UBXII regression’s parameters. The Monte Carlo experiments are performed using the R programming language [30]. Maximization of the log-likelihood function in (11) is carried out using the BFGS quasi-Newton nonlinear optimization algorithm implemented at the optim function available in R. We consider the ordinary least squares estimates (OLSEs) as an initial guess for ***β*** obtained from a linear regression of the transformed responses: *z* = [*g*(*q*_1_), …, *g*(*q*_*n*_)]^⊤^, i.e., the initial point estimate of ***β*** is $\tilde{\beta }={\left(X^{⊤}X\right)}^{-1}X^{⊤}z$. For the shape parameter *c*, we take the same initial guess in Section 4.

The simulations are based on the UBXII regression:

$$\begin{array}{c} \text{logit}\left(q_{i}\right)=\beta_{1}+\beta_{2}x_{i2},\;i=1,\ldots ,n. \end{array}$$ (19)

The covariate ***x***_**2**_ is randomly generated from a standard normal. We combine various values of the parameter vector ***θ*** = (*β*_1_, *β*_2_, *c*)^⊤^ at six different scenarios. The Monte Carlo replications number adopted and the sample sizes considered are the same from Section 4. In each Monte Carlo replication, the inversion method is used to generate *n* occurrences of a random variable *Y*_*i*_∼ UBXII (*q*_*i*_, *c*). By assuming the regression structure defined in Eq (19), it follows that

$$\begin{array}{c} q_{i}=\frac{\text{exp}(\beta_{1}+\beta_{2}\;x_{i2})}{1+\text{exp}(\beta_{1}+\beta_{2}\;x_{i2})}, \end{array}$$

i.e., *q*_*i*_ is equal to the logistic cdf evaluated at (*β*_1_+ *β*_2_ *x*_*i*2_). The statistical quantities computed are also the same of Section 4.

Table 2 presents the results of the Monte Carlo simulations. In general, the RB%s are smaller for larger sample sizes. We can note that the most RB% is equal to 10.02 in scenario four for the smallest sample size, and it refers to the estimate of *c*. For estimates of the parameters *β*_1_ and *β*_2_, all RB%s are below 6.25. In addition, even for *n* = 25, the RMSE values are quite low in any scheme.

**Table 2:** RB%s and RMSEs for the UBXII regression.

Scenario	*β*_1_	*β*_2_	*c*	*n*	RB%			RMSE		
					$\hat{\beta_{1}}$	$\hat{\beta_{2}}$	$\hat{c}$	$\hat{\beta_{1}}$	$\hat{\beta_{2}}$	$\hat{c}$
1	1.3	1.4	2.0	25	−0.3323	0.7091	9.4106	0.1760	0.1499	0.4270
				75	−0.0978	0.3527	2.7208	0.0929	0.0913	0.1975
				150	−0.0392	0.1676	1.2937	0.0644	0.0570	0.1323
				300	−0.0068	0.1248	0.6621	0.0455	0.0451	0.0918
2	0.7	0.4	1.3	25	−1.6493	2.8842	7.8287	0.2680	0.2073	0.2504
				75	−0.1791	1.5523	2.5257	0.1462	0.1397	0.1246
				150	−0.0765	0.8871	1.1396	0.1043	0.0902	0.0843
				300	−0.0671	0.3218	0.5765	0.0732	0.0637	0.0584
3	−0.2	−0.6	1.8	25	6.2548	3.7917	9.8991	0.2599	0.1545	0.4274
				75	1.0153	0.9514	0.1454	0.1454	0.1264	0.1900
				150	0.0641	0.5987	1.3144	0.1001	0.0951	0.1324
				300	0.3399	0.2976	0.6825	0.0724	0.0590	0.0898
4	−0.7	0.4	2.3	25	1.7725	3.1922	10.0246	0.2625	0.2104	0.5838
				75	0.3187	2.0428	3.1417	0.1391	0.1240	0.2780
				150	0.0243	0.7296	1.4848	0.0979	0.0777	0.1894
				300	−0.0512	0.1449	0.7751	0.0691	0.0603	0.1317
5	1.2	−0.5	1.6	25	−0.1732	2.9301	7.9394	0.1860	0.1217	0.3028
				75	−0.0054	0.7715	2.5134	0.1039	0.1069	0.1470
				150	0.0099	0.3839	1.1571	0.0748	0.0663	0.0992
				300	−0.0200	0.2634	0.5976	0.0529	0.0486	0.0687
6	0.4	1.2	2.6	25	−1.4705	0.9087	9.6420	0.1666	0.1364	0.5731
				75	−0.2535	0.4271	2.7730	0.0854	0.0776	0.2673
				150	−0.0936	0.1443	1.3345	0.0597	0.0543	0.1779
				300	0.0397	0.0499	0.6723	0.0431	0.0417	0.1237

**Fig 6:**
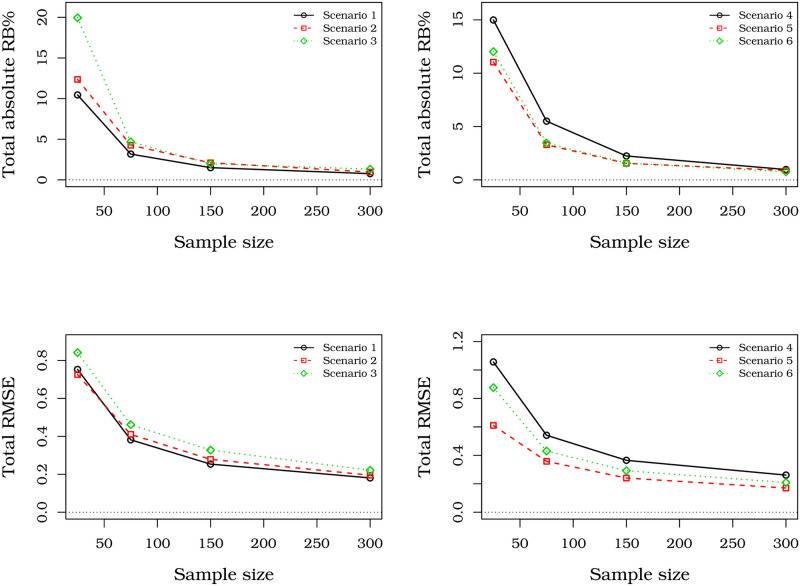
Total absolute RB%s and total RMSE of the MLEs from UBXII regression with different sample sizes.

Fig 6 displays plots for the total RB% and total RMSE versus sample sizes. They reveal that the MLEs are consistent, and their biases quickly tend to zero when the sample size grows. Further, the most RB% is about 20, but it decays to less than 5 to *n* = 75. Thus, as expected, the ML asymptotic properties remain.

We also investigate the behavior of the proposed model competing with the Kumaraswamy (Kw), unit-Weibull (UW) [7], and beta [4] regressions, which are well-known in the analysis of limited data. We aim to compare the performance of the maximum likelihood estimator for estimating the parameters and investigating their performance in case of misspecification of the distribution. We also evaluate the behavior of the AIC and BIC as selection criteria for models from different distributions.

Let *Y* be a random variable Kw distributed under a median-dispersion parameterization [5], say *Y*∼ Kw(*ω*, *d*_*p*_). The pdf of *Y* is

$$\begin{array}{cc} f(y;\omega ,d_{p})= & \;\frac{\;\text{log}\;0.5}{d_{p}\;\text{log}\;\left(1-\omega^{1/d_{p}}\right)}y^{1/d_{p}}{\left(1-y^{1/d_{p}}\right)}^{\;\text{log}\;0.5/\;\text{log}\;(1-\omega^{1/d_{p}})-1},\;y\in \left(0,1\right) \end{array}$$

where 0 < *ω* < 1 is the median of *Y* and *d*_*p*_ > 0 is a dispersion parameter.

The UW quantile regression was recently introduced by [7]. Let *Y*∼ UW(*q*, *γ*) be a random variable having the UW distribution under the parameterization given in [7]. For *y* ∈ (0, 1), the random variable *Y* has density

$$\begin{array}{c} f\left(y;q,\gamma \right)=\frac{\gamma }{y}(\frac{\;\text{log}\;\tau }{\;\text{log}\;q}){\left(\frac{\;\text{log}\;y}{\;\text{log}\;q}\right)}^{\gamma -1}\tau^{{\left(\;\text{log}\;y/\;\text{log}\;q\right)}^{\gamma }}, \end{array}$$

where 0 < *q* < 1 is the *τ*th quantile, *γ* > 0 is a shape parameter, and *τ* ∈ (0, 1) is assumed known. Here, it will be considered that *τ* = 0.5 in order to model the median of *Y*.

[4] pioneered the beta regression. Different parameterizations can be considered for the beta distribution. We consider the mean-precision based parameterization. Let *Y* be a random variable that follows a beta distribution, say *Y*∼ Beta(*μ*, *ϕ*). For *y* ∈ (0, 1), the *Y* density is

$$\begin{array}{c} f\left(y;\mu ,\phi \right)=\frac{\Gamma (\phi )}{\Gamma (\mu \phi )\Gamma ((1-\mu )\phi )}y^{\mu \phi -1}{\left(1-y\right)}^{(1-\mu )\phi -1}, \end{array}$$

where 0 < *μ* < 1 is the mean of *Y*, *ϕ* > 0 is a precision parameter and $\Gamma \left(\alpha \right)=\int_{0}^{\infty }x^{\alpha -1}e^{-x}dx$ is the complete gamma function. Under this parameterization the variance of *Y* is *V*(*μ*)/(1+ *ϕ*), with *V*(*μ*) = *μ*(1 − *μ*).

The regression structure for the Kw, UW, and beta distributions is analogous to (19). The main differences are the assumptions under the random components and modeled location parameters. To get the Kw regression, *q* must be replaced by the median (*ω*) in Eq (19) and supposed that *Y*_*i*_∼ Kw(*ω*_*i*_, *d*_*p*_). The UW regression is obtained by considering the structure (19) and assuming that *Y*_*i*_∼ UW(*q*_*i*_, *γ*). In the beta regression, the location parameter is the mean (*μ*). Hence, in Eq (19), *q* must be replaced by *μ* and supposed that *Y*_*i*_∼ Beta(*μ*_*i*_, *ϕ*). Thus, considering these regression structures, the simulation was performed in the following steps.

1. We generate a sample with *n* = 100 observations for each regression. The parameter values were selected from Scenario 1 in Table 2, replacing *c* for the respective shape parameter.
2. We fit the true regression and the UBXII regression for each generating scheme. When the UBXII is the true model, we fit all the competitors.
3. We compute the MSE, AIC, and BIC for all fitted models.
4. For each scenario considered, 5,000 replications were performed.
5. For the MSE, we compute the average of all replications. For the AIC and BIC, the frequencies (%) of correct model selection are computed.

Table 3 displays the performance of the UBXII model when compared with the existing ones. The estimate $\hat{\theta }$ is $\hat{c}$ for UBXII, ${\hat{d}}_{p}$ for Kw, $\hat{\gamma }$ for UW, and $\hat{\phi }$ for beta regression. We observe that the estimates obtained with the Beta and KW differ from those from the UBXII and UW distributions. The last two present estimates are close to each other. It shows that the traditional beta and Kw densities were not suitable to describe the data generated from the UBXII. The UW is the most competitive model but still presents a worse performance for fitting UBXII random variables. Concerning the model selection approaches, all measures were able to select the correct model. The results for AIC and BIC were very similar, their success rates exceeding 92% for all generating schemes. Thus, the information criteria are reliable for model selection among the considered competitive models.

**Table 3:** Results of Monte Carlo simulations for Scenario 1 (*β*_1_ = 1.3, *β*_1_ = 1.4, and *c* = 2.0), with *n* = 100 and *R* = 5000 replications and MSE mean, AIC and BIC frequencies (%) of correct model selection.

Simulation	UBXII(*q*_*i*_, *c*)				Kw(*ω*_*i*_,*d*_*p*_)		UW(*q*_*i*_, *γ*)		Beta(*μ*_*i*_, *ϕ*)	
Par./Meas.	UBXII	Kw	UW	Beta	UBXII	Kw	UBXII	UW	UBXII	Beta
${\hat{\beta }}_{1}$	1.2984	0.7471	1.2972	0.9241	2.0396	1.3267	1.3238	1.2986	3.2141	1.2071
	(0.0802)	(0.4013)	(0.0949)	(0.1798)	(0.3435)	(0.2944)	(0.0752)	(0.0732)	(0.3661)	(0.1245)
${\hat{\beta }}_{2}$	1.4040	1.0398	1.5873	1.2794	0.9411	1.4311	1.3597	1.4034	2.4634	1.2125
	(0.0670)	(0.2549)	(0.1246)	(0.2181)	(0.2664)	(0.2545)	(0.0624)	(0.0603)	(0.3905)	(0.1105)
$\hat{\theta }$	2.0441	1.9806	1.3982	6.7714	0.4688	1.9139	2.1814	2.0451	0.4025	1.9896
	(0.1647)	(3.9387)	(0.3370)	(2.2466)	(0.0542)	(0.3801)	(0.1777)	(0.1661)	(0.0463)	(0.3092)
AIC (%)	92.1600	0.0000	7.8400	0.0000	0.0400	99.9600	3.6600	96.3400	0.0200	99.9800
BIC (%)	92.1600	0.0000	7.8400	0.0000	0.0400	99.9600	3.6600	96.3400	0.0200	99.9800
MSE	0.0053	1.0608	0.0136	0.3587	4.6296	1.0705	0.0044	0.0035	1.5143	0.0209

## 6 Application

In this section, we assess the UBXII regression performance on real data. The analysis is carried out using the R statistical computing environment [30]. We fit the UBXII regression and compare it with the Kw, UW [7], and beta [4] regressions, which are well-known in the analysis of limited data and were also considered in the simulation experiment. The R codes of the simulation studies and application are available at https://github.com/tatianefribeiro/UBXII_regression. We get the data from the higher education census conducted yearly by the Brazilian National Institute for Educational Studies and Research “Anísio Teixeira”. We are interested in the dropout proportion for animal sciences courses and factors associated with their enrollment and organizational structure. However, the response variable is not directly obtained from the original data set, and we use mining data techniques to obtain it from other reported variables. After preprocessing and cleaning steps, we select 40 covariates as possible predictors. A detailed description of the data mining tools employed and the final data set are available in Supporting information.

The UBXII, Kw, UW, and beta regressions also are used as data mining tools to select a subset of predictors that properly fits the dropout proportion. We test several combinations of predictors using the measures described in Section 6.3. to define the final regressions on each class. In what follows, we describe the response variable and predictive covariates used in our regression analysis.

The response variable is the dropout proportion from 2009 until 2017 of 77 Brazilian undergraduate animal sciences courses. For each course *i* (*i* = 1, …, 77), we consider three covariates as follows: i) quantity of vacancies offered in the morning shift, denoted by *x*_*i*2_; ii) a dummy variable that equals one if the course guarantees conditions of accessibility for people with disabilities, and zero otherwise, denoted by *x*_*i*3_; and iii) a dummy variable, denoted by *x*_*i*4_, that equals one if the course works on the night shift, and zero otherwise.

Let ***y*** = (*y*_1_, …, *y*_77_)^⊤^ be the vector of the response variable and ***X*** = (***x***_1_, …, ***x***_4_) the covariates matrix, where ***x***_1_ is a vector column with 77 ones and ***x***_*j*_ = (*x*_1*j*_, …, *x*_77*j*_)^⊤^, with *j* = 2, …, 4. Table 4 provides a descriptive summary of the response variable (***y***) and quantitative covariate (***x***_**2**_), revealing that ***y*** has negatively skewed distribution and lighter tails than a normal distribution. Further, its mean is close to the median, the standard deviation (SD) is low, and the values range is sizeable because the minimum and maximum are 0.1077 and 0.9714, respectively. The covariate *x*_2_ presents different degrees of variability, skewness, and kurtosis.

**Table 4:** Descriptive statistics from the response variable and quantitative covariates.

Var.	Statistics						
	Mean	Median	SD	Skewness	Kurtosis	Min.	Max.
***y***	0.5736	0.5965	0.1818	−0.3449	0.0854	0.1077	0.9714
***x***_2_	13.7532	0.0000	29.5449	2.0533	3.2902	0.0000	120.0000

To study the covariates’ effects on the median dropout proportion, we set *τ* = 0.5 and specify the UBXII regression as

$$\begin{array}{c} \text{logit}\left(q_{i}\right)=\eta_{i}=\beta_{1}+\beta_{2}\;x_{i2}+\beta_{3}\;x_{i3}+\beta_{4}\;x_{i4}, \end{array}$$

For comparison purposes, we also fit the Kw, UW, and beta regressions considering the same covariates combination and link function.

**Fig 7:**
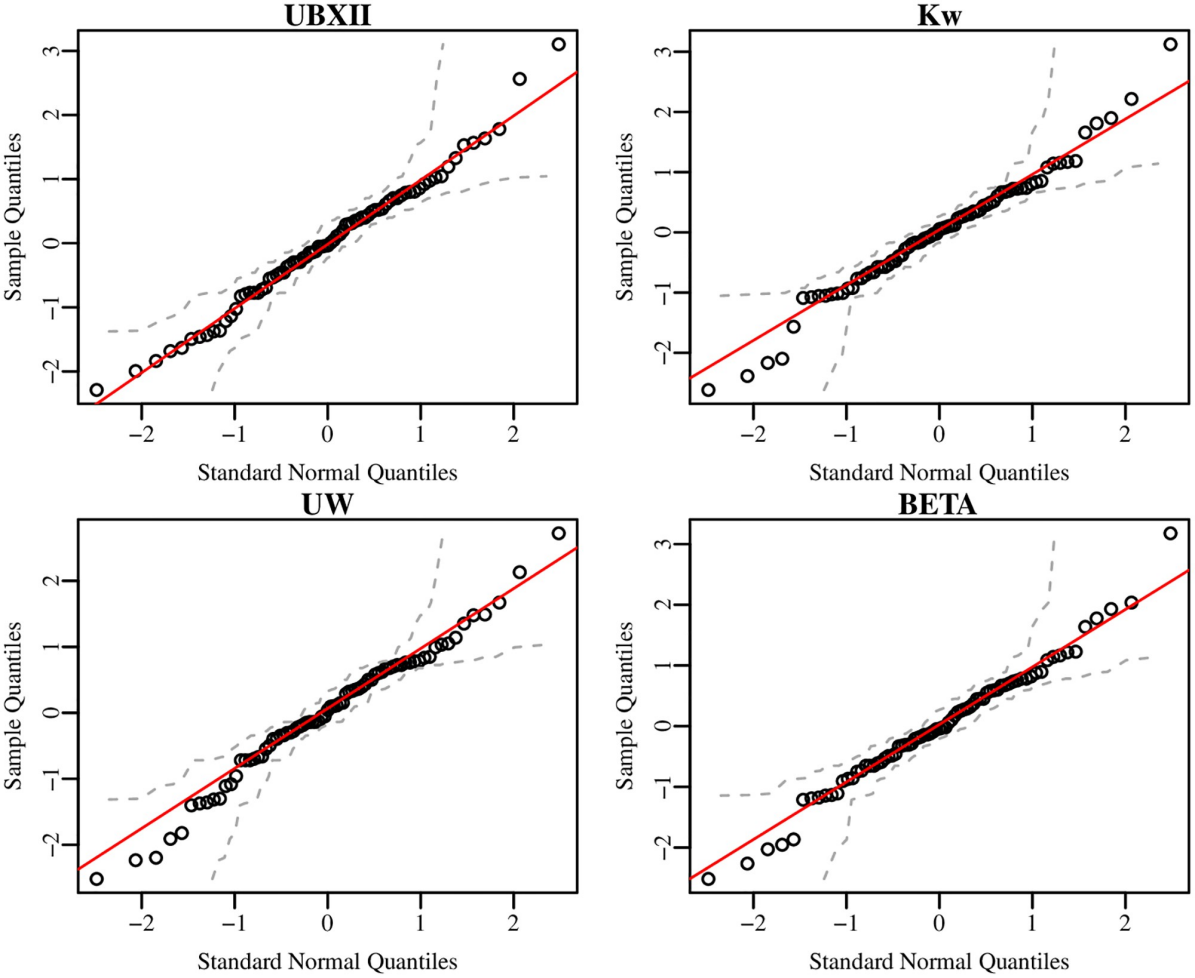
QQ-plots of the UBXII, Kw, UW, and beta regressions’ residuals.

Table 5 brings some goodness-of-fit measures such as AIC, BIC, and $R_{G}^{2}$, the *p*-values of the Anderson-Darling test (AD) [31] to validate the null hypothesis that errors are normally distributed, the *p*-values from RESET-like test (RES), and the statistic obtained from the LOOCV approach (CV_(77)_) that allows assessing the prediction performance of the fitted regressions. We consider *α* = 0.05 as a significance level for all performed hypothesis tests. According to the RESET-like tests, all models are correctly specified. Similarly, the *p*-values from Anderson-Darling tests indicate is reasonable supposing normality of the errors at each class. It is noteworthy that most of some goodness-of-fit measures suggest that the UBXII regression is more suitable to fit the dropout proportion in the Brazilian zootechnics course between 2009 and 2017 than other considered class of regressions. Moreover, the *CV*_(77)_ estimate for the fitted UBXII regression is the smallest among all other fitted regressions. This means that the proposed regression leads to better predictions than the classical regressions used in the context of restricted response to the unit interval. Indeed, in Fig 7 it is possible to note that the UBXII regression provides the best fit for this data set since about 97% of the points are under the red line in the QQ-plot of fitted UBXII regression’s residuals.

**Table 5:** Goodness-of-fit measures and LOOCV statistic for the fitted regressions.

Regression	AIC	BIC	R${}_{G}^{2}$	AD	RES	CV_(77)_
UBXII	−55.8423	−44.1233	0.2348	0.8229	0.4334	0.0259
Kw	−48.8064	−37.0873	0.1898	0.2765	0.8354	0.0260
UW	−52.6329	−40.9139	0.2565	0.2795	0.5764	0.0266
BETA	−52.0595	−40.3405	0.2235	0.5433	0.8383	0.0285

In Table 6, we provide the estimates of the parameters, standard errors, *t* statistic value, and *p*-values for the UBXII regression. Results from other fitted regressions are given in Supporting information; see Table 2. The effect of the three considered covariates under the response’s median is positive. Further, according to the estimate of *β*_4_, the covariate *x*_*i*4_ presents the most impact on the median. That is, the odds ratio increases substantially if the course works on the night shift. This result may be related to the fact that many of the night students need to work during the day, making it challenging to persist [32]. However, the offer of night courses results from conquests achieved by popular pressure to meet the requirements of a population mainly consisting of workers [33]. Thus, our finding raises the discussion on the need to provide a better service to this public. For example, the low offer of extracurricular activities for evening students is one of the problems reported by [33].

**Table 6:** Fitted UBXII regression for the dropout proportion in the Brazilian zootechnics course.

Parameter	Estimate	Std. Error	t value	*p*-value
*β*_1_	−0.0509	0.1294	−0.3932	0.6953
*β*_2_	0.0082	0.0024	3.4429	0.0010
*β*_3_	0.5389	0.1560	3.4535	0.0009
*β*_4_	0.8310	0.2665	3.1183	0.0026
*c*	2.3780	0.2032	—	—

**Fig 8:**
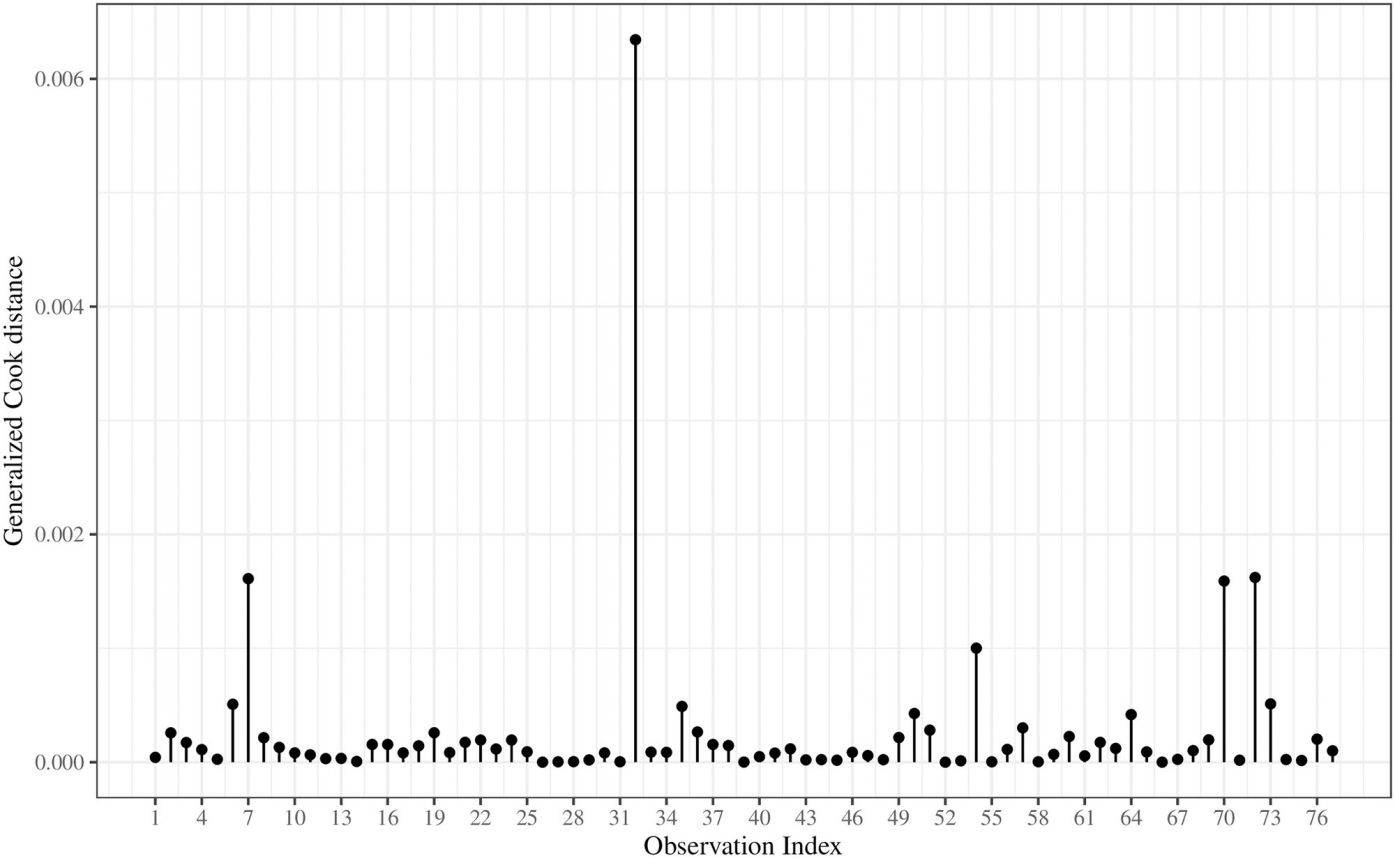
Generalized Cook distance for the UBXII regression.

**Fig 9:**
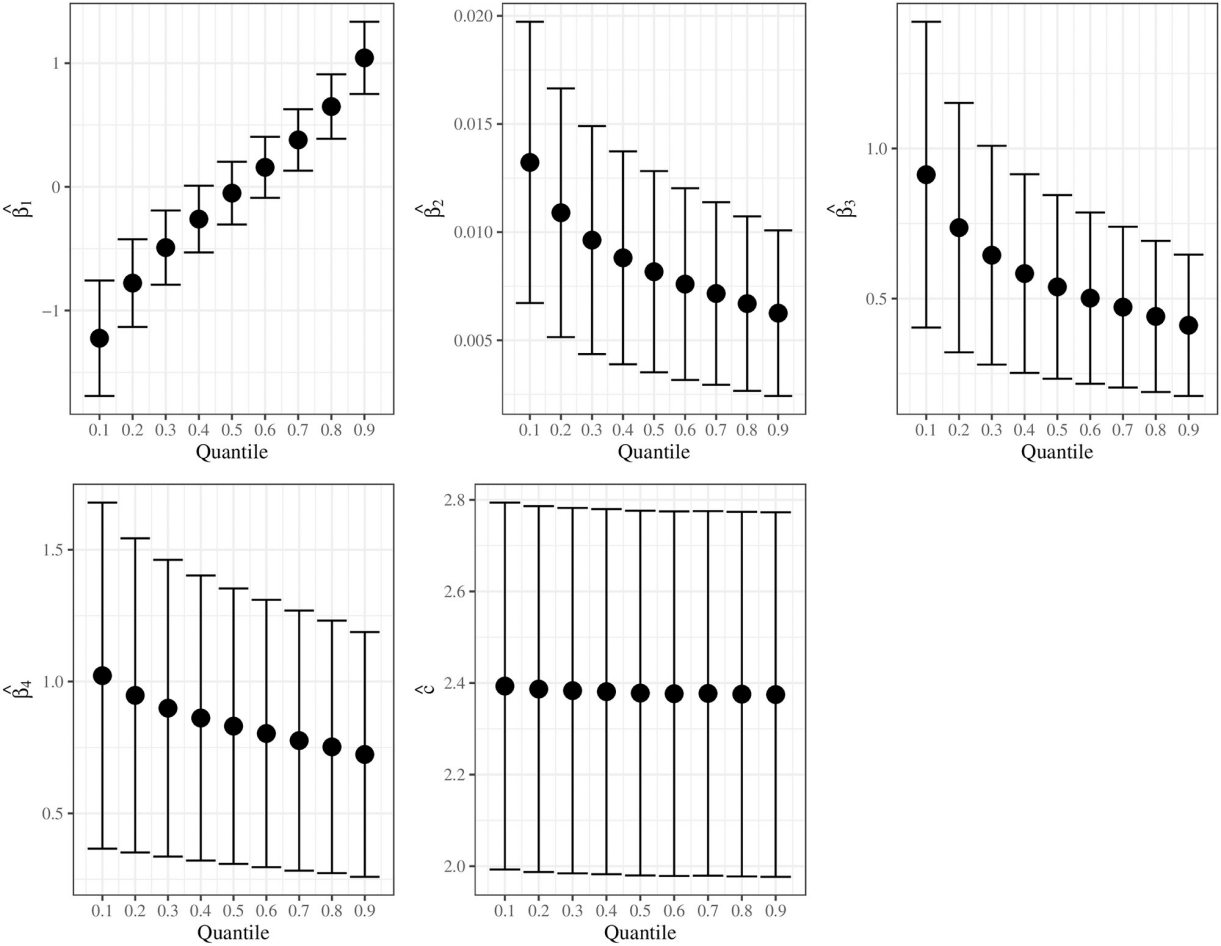
Parameter estimates and the 95% pointwise confidence intervals for the UBXII regression by considering *τ* ∈ {0.1, 0.2, …, 0.9}.

Fig 8 plots the GD for the UBXII regression. We can note that only observation 32 highlights the others. It corresponds to the *Faculdade de Estudos Superiores de Minas Gerais* and, with dropout proportion of 0.8163, is upper the 3th quartile of the data set. However, it is not potentially influential since the GD associated is smaller than 4/*n*. Fig 9 assesses the impact of different *τ* values on the parameter estimates. We compute the 95% confidence intervals and point estimates for the UBXII regression by considering *τ* ∈ {0.1, 0.2, …, 0.9}. We observe that the intercept estimates become higher as *τ* increases, and the other regression coefficients are negatively related to the quantiles. It indicates that the covariates have a more substantial impact on explaining smaller quantiles of the dropout proportion. Finally, $\hat{c}$ does not seem to be affected by variations of *τ* values. It is worth noting that similar behavior is reported by [7] for the shape parameter of the UW regression.

## 7 Conclusions

We define a new unit quantile regression based on an alternative reparametrization for the unit Burr XII (UBXII) distribution pioneered by [8]. A highlight of the proposed parametrization is that one of its parameters, *q*(*τ*), represents the *τ*th quantile of the random variable. The researcher defines the *τ* value and assumes a regression structure on *q*(*τ*). We investigated some additional statistical quantities to those explored by [8], namely the score functions, and observed information matrix. The maximum likelihood method is used for parameter estimation, and Monte Carlo simulations show that its properties remain. We adapt several diagnostic analysis and model selection techniques that can be employed to check the goodness-of-fit of the estimated model.

The utility of the proposed regression is illustrated with an application that targeted to explain the linear relation between the dropout proportion of Brazilian undergraduate animal sciences courses and some factors associated with their enrollment and organizational structure. An essential aspect of quantile-based regression is the possibility of separately analyzing the covariates’ marginal effect on each response’s quantile. That allowed us to find that the effects of some factors, such as the number of vacancies, accessibility, and night shift, are more negligible on courses with fewer dropouts (those belonging to the lower quantiles). Another notable result is the positive effect between courses with night shifts and the dropout rate. This phenomenon is explained by the work carried out by the students during the morning shift, which makes persistence difficult. This situation must be considered by those who make educational policies since the opening of vacancies in the night shift must also be complemented by student attendance policies.

Additionally, we also fit the data set using other well-known regression models, such as the Kumaraswamy, unit-Weibull, and beta. The fit of the UBXII regression is superior to all of them since it provides better prediction performance. Thus, the UBXII regression is an alternative quite competitive for modeling data restricted to the unit interval and can be applied when the classical regressions are not unsuitable. That feature of capturing the nature of double-bonded variables makes the new model have a wide range of applications; for example, in the educational area, it can be helpful for modeling educational indicators such as graduation and persistence proportions of undergraduate and postgraduate courses. It may also be an alternative to educational measurements from different countries, such as in the applications [13–15], and [16] provided.

We end with some comments on possible future work. It should be noted that in conventional regression modeling, the non-existence of serial correlation between errors is assumed. In that sense, an extension of our proposal is the development of models that consider exogenous covariates in the median response with an Autoregressive Moving Average structure to handle serial dependence. It is important to highlight that the UBXII regression can be extended to the neutrosophic statistics analysis. This kind of analysis is applied when data or a part of it are indeterminate; that is, data have uncertain observations. Recently, some studies have been done in this context. [34] introduced the neutrosophic analysis of variance to test teaching methods using data collected from university students. [35] proposed a new Z-test for uncertainty events under neutrosophic statistics, which was applied to the Covid-19 data. In the regression context, [36] concluded that it is preferable to use Neutrosophic multiple regression over the classical regression models since this method is the most efficient for forecast the uncertainty observation data.

## Supporting information

S1 File Supplement to “The unit Burr XII regression: Properties, Simulation and application”. It provides a detailed description of the data mining tools employed to obtain the final data set used in the application study in Section 7 and results from Kw, UW, and beta fitted regressions to the dropout proportion of Brazilian animal science courses.
(PDF)

S1 Data Droupot proportion of Brazilian animal science courses data set. Data set used in the application study in Section 7.
(ODS)

S1 Appendix (PDF)
