# Supplementary material for: Another unit Burr XII quantile regression model based on the different reparameterization applied to dropout in Brazilian undergraduate courses
Source: PLoS One. 2022 Nov 3;17(11):e0276695. doi: 10.1371/journal.pone.0276695 (PMC9632897; doi:10.1371/journal.pone.0276695)
Supplement: S1 File — It provides a detailed description of the data mining tools employed to obtain the final data set used in the application study in Section 7 and results from Kw, UW, and beta fitted regressions to the dropout proportion of Brazilian animal science courses. (PDF) [file pone.0276695.s001.pdf]

The unit Burr XII regression applied to dropout in Brazilian undergraduate courses

Tatiane F. Ribeiro[1*], Gauss M. Cordeiro[2], Fernando A. Peña-Ramírez[3], Renata R. Guerra[3]

1 Instituto de Matemática e Estatística, Universidade de São Paulo, São Paulo/SP, Brazil
2 Departamento de Estatística, Universidade Federal de Pernambuco, Recife/PE, Brazil
3 Departamento de Estatística, Universidade Federal de Santa Maria, Santa Maria/RS, Brazil

* tatianefr@ime.usp.br

Supporting information

We provide a supplementary material that contains information to extract the full database and explains the methodology used in preprocessing and cleaning step. Further, we present a table with the description of the data set's variables used in the application and a table with the results from other fitted Kw, UW, and beta regressions for the considered data set.

Data extraction

The data for this study were otained from the publicly-available higher education census (HEC) microdata. Since 1995, the HEC is conducted yearly by the Brazilian National Institute for Educational Studies and Research "Anísio Teixeira" (INEP) and the data are available at `http://portal.inep.gov.br/web/guest/microdados`. It contains information about the Brazilian higher education system divided into four microdata files, each one presenting students, course, professors and education institution variables. Those files are defined as follows:

1. DM_IES: composed by higher education institutions (HEIs) variables such as the institution's code, administrative category, city, and federation unit, among others;
2. DM_CURSO: contains variables about the undergraduate courses such as the course workload, shift (morning, afternoon, night), number of vacancies, among others;
3. DM_ALUNO: contains variables related to the students such as socio-demographic information from the students, course, admision form, among others;
4. DM_DOCENTE: provides variables related to the professors linked to each HEI, such as socio-demographic and career informations, among others.

We are interested in the dropout proportion for animal sciences courses and factors associated with their enrollment and organizational structure. The DM_ALUNO file provides the information to construct the dropout proportion. The other variables are obtained from the DM_IES and DM_CURSO files. The following section describes the data mining tools employed to obtain the final data set.

Preprocessing and cleaning

Data preprocessing and cleaning involves basic operations to collect and filter the necessary information in order to conduct desired statistical analysis. We perform the data filtering in the R programming language [1]. We use the `ffbase` [2], `tidyverse` [3], and `dplyr` [4] packages, necessary to treat a big database. The population is the cohort of the freshmen animal sciences students in academic year 2009. Each of them has a related unique identification code in the DM_ALUNO file, which allows us to follow them up until 2017, or until the dropout/graduate outcome. The variable CO_ALUNO_SITUACAO identify the student's situation in each census. It is from this variable that we build the dropout proportion.

From the CO_ALUNO_SITUACAO variable, we reclassify the students according to their last register in the census and construct a new variable under the following categories

1. **dropout**: if the student transferred to another course of the same HEI or who detached from the course, originally encoded as 4 and 5, respectively;

2. **graduate**: if the student completed its undergraduate study, originally encoded as 6;

3. **censoring**: students who have situation likely forming, attending, locked enrollment, or deceased, originally encoded as 1, 2, 3, and 7, respectively.

The response variable for the ith course is given by

$$\text{DROPOUT\_PROPORTION}_i = \frac{\text{number of students with dropout outcome in the } i\text{th course}}{\text{number of students with dropout or graduate outcome in the } i\text{th course}},$$

where $i = 1, \ldots, 78$. The students classified as censoring are not considered since none outcome is observed in this group and one course is eliminated since it had no graduated students until 2017. Thus, we obtain 77 observations corresponding to the dropout proportions of Brazilian animal sciences courses with freshmen students in 2009. We join the organizational variables, from the DM_IES and DM_CURSO files in the HEC of 2009, with the dropout proportion. Finally, we select and clean the those covariates to obtain the final data set. In the cleanning process we i) eliminate some variables with missing observations and identification codes; and ii) join some variables to perform data reduction. The final data set contains the dropout proportion and other 42 covariates.

Table 1 provides the nomenclature (nom.) and a brief description of the response variable, and covariates of the final data set.

Table 1. Response variable and covariates with its respective description

Nom.	Variable	Description
Y	DROPOUT_PROPORTION (response variable)	Dropout proportion from 2009 until 2017 of Brazilian undergraduate animal sciences courses.
$\boldsymbol{x}_1$	ID[1]	Name of the university to which the course belongs.
$\boldsymbol{x}_2$	QT_VAC_MORNING	Quantity of vacancies offered in the morning shift.
$\boldsymbol{x}_3$	IN_ACCESSIBILITY	Dummy variable that equals one if the course guarantees conditions of accessibility for people with disabilities, and zero otherwise.
$\boldsymbol{x}_4$	IN_NIGHT_COURSE	Dummy variable that equals one if the course works on the night shift, and zero otherwise.
$\boldsymbol{x}_5$	IN_LIBRAS_TRANSLATOR	Dummy variable that equals one if the course provides a translator of Brazilian sign language interpreter (LIBRAS), and zero otherwise.
$\boldsymbol{x}_6$	IN_HIGH_RELIEF	Dummy variable that equals one if the course offers adaptation to high relief of graphics, engravings and figures, and zero otherwise.
$\boldsymbol{x}_7$	IN_AUDIO	Dummy variable that equals one if the course has material in audio, and zero otherwise.
$\boldsymbol{x}_8$	IN_BRAILLE	Dummy variable that equals one if the course has material in Braille, and zero otherwise.
$\boldsymbol{x}_9$	IN_ENL_CHARACTER	Dummy variable that equals one if the course offers material with enlarged characters, and zero otherwise.
$\boldsymbol{x}_{10}$	IN_LIBRAS_DISCIPLINE	Dummy variable that equals one if the course provides translator of LIBRAS, and zero otherwise.
$\boldsymbol{x}_{11}$	IN_GUIDE_INTERPRETER	Dummy variable that equals one if the course makes available guides-interpreter, and zero otherwise.
$\boldsymbol{x}_{12}$	IN_LIBRAS_MATERIAL	Dummy variable that equals one if the course has material in LIBRAS, and zero otherwise.
$\boldsymbol{x}_{13}$	IN_SPEECH_SYNTHESIS	Dummy variable that equals one if the course offers a speech synthesis, and zero otherwise.
$\boldsymbol{x}_{14}$	IN_MORNING_COURSE	Dummy variable that equals one if the course works on the morning shift, and zero otherwise.
$\boldsymbol{x}_{15}$	IN_OTHER_ADM_FORMS	Dummy variable that equals one if the course has alternative forms of admission in addition to the regular ones, and zero otherwise.
$\boldsymbol{x}_{16}$	IN_EVENING_COURSE	Dummy variable that equals one if the course works on the evening shift, and zero otherwise.
		(It continues)

[1]Identification variable not considered for modeling.

		(Continuation)
$\boldsymbol{x}_{17}$	IN_AGREEMENT	Dummy variable that equals one if the course makes available enter through course of agreement for foreign students, and zero otherwise.
$\boldsymbol{x}_{18}$	IN_DIST_LEARNING	Dummy variable that equals one if the classroom course offers distance learning, and zero otherwise.
$\boldsymbol{x}_{19}$	IN_USE_LAB	Dummy variable that equals one if the course uses the laboratories from the HEI, and zero otherwise.
$\boldsymbol{x}_{20}$	NU_COURSE_LOAD	Course load
$\boldsymbol{x}_{21}$	NU_TIME_COMP	Minimum time to complete the course in number of semesters
$\boldsymbol{x}_{22}$	QT_VAC_INTEGRAL	Quantity of vacancies offered in the integral shift.
$\boldsymbol{x}_{23}$	QT_VAC_NIGHT	Quantity of vacancies offered in the night shift.
$\boldsymbol{x}_{24}$	QT_VAC_EVENING	Quantity of vacancies offered in the evening shift.
$\boldsymbol{x}_{25}$	QT_SEL_PROCESS	Number of students who entered in the course through selection process.
$\boldsymbol{x}_{26}$	QT_SEL_OTHER_FORM	Number of students who entered in the course through other selection forms.
$\boldsymbol{x}_{27}$	IN_CAPITAL_HEI	Dummy variable that equals one if the HEI to which the course belongs is located in the capital, and zero otherwise.
$\boldsymbol{x}_{28}$	IN_ADM_CAT_1	Dummy variable that equals one if the HEI to which the course belongs has a federal administrative category, and zero otherwise.
$\boldsymbol{x}_{29}$	IN_ADM_CAT_2	Dummy variable that equals one if the HEI to which the course belongs has a state administrative category, and zero otherwise.
$\boldsymbol{x}_{30}$	IN_ADM_CAT_3	Dummy variable that equals one if the HEI to which the course belongs has a municipal administrative category, and zero otherwise.
$\boldsymbol{x}_{31}$	IN_ADM_CAT_4	Dummy variable that equals one if the HEI to which the course belongs has a private in the strict sense administrative category, and zero otherwise.
$\boldsymbol{x}_{32}$	IN_ACADEM_ORG_1	Dummy variable that equals one if the HEI's academic organization to which the course belongs is university, and zero otherwise.
$\boldsymbol{x}_{33}$	IN_ACADEM_ORG_2	Dummy variable that equals one if the HEI's academic organization to which the course belongs is university center, and zero otherwise.

Table 2 brings the estimates and p-values of the fitted Kw, UW, and beta regressions for the dropout proportion in the Brazilian zootechnics courses between 2009 and 2017.

		(Continuation)
$\boldsymbol{x}_{34}$	IN_ACADEM_ORG_3	Dummy variable that equals one if the HEI's academic organization to which the course belongs is college, and zero otherwise.
$\boldsymbol{x}_{35}$	QT_TEC_INCOMP_ELEM	Number of technical-administrative employees of the HEI (of which the ith course is part) with incomplete elementary education.
$\boldsymbol{x}_{36}$	QT_TEC_HIGH_SCHOOL	Number of technical-administrative employees of the HEI (of which the ith course is part) with high school.
$\boldsymbol{x}_{37}$	QT_TEC_HIGHER_EDUC	Number of technical-administrative employees of the HEI (of which the ith course is part) with higher education.
$\boldsymbol{x}_{38}$	QT_TEC_SPEC	Number of technical-administrative employees of the HEI (of which the ith course is part) with specialization.
$\boldsymbol{x}_{39}$	QT_TEC_MASTER	Number of technical-administrative employees of the HEI (of which the ith course is part) with master's education.
$\boldsymbol{x}_{40}$	QT_TEC_DOC	Number of technical-administrative employees of the HEI (of which the ith course is part) with a doctorate.

Table 2. Estimates and p-values of the fitted Kw, UW, and beta regressions for the dropout proportion in the Brazilian zootechnics courses.

Parameter	Kw		UW		Beta	
	Estimate	p-value	Estimate	p-value	Estimate	p-value
β_1	0.0379	0.7251	−0.1778	0.2102	−0.0595	0.5954
β_2	0.0067	0.0378	0.0098	0.0004	0.0082	0.0046
β_3	0.4401	0.0095	0.6801	0.0002	0.5137	0.0023
β_4	0.7169	0.1139	0.9107	0.0055	0.8152	0.0397
d_p, γ, ϕ	0.3116	< 0.0001	1.9202	< 0.0001	7.5636	< 0.0001

References

1. R Core Team. R: A Language and Environment for Statistical Computing; 2019.
2. de Jonge E, Wijffels J, van der Laan J. ffbase: Basic Statistical Functions for Package 'ff'; 2020.
3. Wickham H, Averick M, Bryan J, Chang W, McGowan LD, François R, et al. Welcome to the tidyverse. Journal of Open Source Software. 2019;4:1686.
4. Wickham H, François R, Henry L, Müller K. dplyr: A Grammar of Data Manipulation; 2020.
