# Supplementary material for: Another unit Burr XII quantile regression model based on the different reparameterization applied to dropout in Brazilian undergraduate courses
Source: PLoS One. 2022 Nov 3;17(11):e0276695. doi: 10.1371/journal.pone.0276695 (PMC9632897; doi:10.1371/journal.pone.0276695)
Supplement: S1 Appendix — (PDF) [file pone.0276695.s003.pdf]

Appendix A

Initially, this appendix provides the observed information matrix for the unit Burr XII (UBXII) distribution and a detailed calculation of the observed information matrix for the UBXII regression, defined in Section 5.

Observed information matrices

In what follows, we present the second-order derivatives of the log-likelihood function to the parameters vector $\boldsymbol{\theta}$, of the UBXII distribution. The elements of the matrix $J(\boldsymbol{\theta})$ presented in Section 3 are

$$
\begin{aligned}
U_{cc} = &-\frac{n}{c^2} - \frac{n\log^2(\log q^{-1})[t(q)-1]}{t(q)\log[t(q)]} + \frac{n\log^2(\log q^{-1})\log^{2c} q^{-1}}{[t(q)]^2\log[t(q)]} + \frac{n\log^2(\log q^{-1})\log^{2c} q^{-1}}{[t(q)]^2\log^2[t(q)]} \\
&- \sum_{i=1}^{n}\frac{\log^2(\log y_i^{-1})[t(y_i)-1]}{[t(y_i)]^2} - \frac{\log\tau^{-1}}{\log[t(q)]}\left[\sum_{i=1}^{n}\frac{\log^2(\log y_i^{-1})\log^c y_i^{-1}}{t(y_i)}\right. \\
&\left. - \sum_{i=1}^{n}\frac{\log^2(\log y_i^{-1})\log^{2c} y_i^{-1}}{[t(y_i)]^2}\right] + \frac{2\log(\log q^{-1})\log\tau^{-1}[t(q)-1]}{t(q)\log^2[t(q)]} \\
&\times \sum_{i=1}^{n}\frac{\log(\log y_i^{-1})[t(y_i)-1]}{t(y_i)} + \left\{\frac{\log^2(\log q^{-1})\log\tau^{-1}[t(q)-1]}{t(q)\log^2[t(q)]}\right. \\
&\left. - \frac{\log^2(\log q^{-1})\log\tau^{-1}\log^{2c} q^{-1}}{[t(q)]^2\log^2[t(q)]} - \frac{2\log^2(\log q^{-1})\log\tau^{-1}\log^{2c} q^{-1}}{[t(q)]^2\log^3[t(q)]}\right\}\sum_{i=1}^{n}\log[t(y_i)],
\end{aligned}
$$

$$
\begin{aligned}
U_{qq}(\boldsymbol{\theta}) = &\frac{n\,c^2\log^{2c-2} q^{-1}}{q^2\,[t(q)]^2\log[t(q)]} + \frac{n\,c^2\log^{2c-2} q^{-1}}{q^2\,[t(q)]^2\log^2[t(q)]} - \frac{n\,c\,(c-1)\log^{c-2} q^{-1}}{q^2\,t(q)\log[t(q)]} \\
&- \frac{n\,c\log^{c-1} q^{-1}}{q^2\,t(q)\log[t(q)]} + \left[\frac{c\,(c-1)\log\tau^{-1}\log^{c-2} q^{-1}}{q^2\,t(q)\log^2[t(q)]} + \frac{c\log\tau^{-1}\log^{c-1} q^{-1}}{q^2\,t(q)\log^2[t(q)]}\right. \\
&\left. - \frac{2\,c^2\log\tau^{-1}\log^{2c-2} q^{-1}}{q^2\,[t(q)]^2\log^3[t(q)]} - \frac{c^2\log\tau^{-1}\log^{2c-2} q^{-1}}{\{q\,t(q)\log[t(q)]\}^2}\right]\sum_{i=1}^{n}\log[t(y_i)],
\end{aligned}
$$

and

$$
\begin{aligned}
U_{cq}(\boldsymbol{\theta}) =& \frac{n\,c\log(\log q^{-1})\log^{c-1}q^{-1}}{q\,t(q)\log[t(q)]} + \frac{n\log^{c-1}q^{-1}}{q\,t(q)\log[t(q)]} - \frac{n\,c\log(\log q^{-1})\log^{2c-1}q^{-1}}{q\,[t(q)]^2\log[t(q)]} \\
& - \frac{n\,c\log(\log q^{-1})\log^{2c-1}q^{-1}}{q\,[t(q)]^2\log^2[t(q)]} - \frac{c\log\tau^{-1}\log^{c-1}q^{-1}}{q\,t(q)\log^2[t(q)]}\sum_{i=1}^{n}\frac{\log(\log y_i^{-1})\log^c y_i^{-1}}{t(y_i)} \\
& + \left[\frac{2\,c\log(\log q^{-1})\log\tau^{-1}\log^{2c-1}q^{-1}}{q\,[t(q)]^2\log^3[t(q)]} + \frac{c\log(\log q^{-1})\log\tau^{-1}\log^{2c-1}q^{-1}}{q\,[t(q)]^2\log^2[t(q)]}\right. \\
& \left. - \frac{\log\tau^{-1}\log^{c-1}q^{-1}}{q\,t(q)\log^2[t(q)]} - \frac{c\log(\log q^{-1})\log\tau^{-1}\log^{c-1}q^{-1}}{q\,t(q)\log^2[t(q)]}\right]\sum_{i=1}^{n}\log[t(y_i)].
\end{aligned}
$$

Next, we obtain the score function and observed information matrix for the parameter vector $(\boldsymbol{\beta}^\top, c)^\top$ from the regression (10). First, we obtain the components of the score vector $\boldsymbol{U}$ in Section 5.1. Notice that $U_{\boldsymbol{\beta}} = [U_{\beta_1}(\boldsymbol{\beta}, c), \ldots, U_{\beta_k}(\boldsymbol{\beta}, c)]^\top$ is the first component of $\boldsymbol{U}$. Invoking the chain rule, we have

$$
U_{\beta_j} \equiv \frac{\partial \ell(\boldsymbol{\beta}, c)}{\partial \beta_j} = \sum_{i=1}^{n}\left[\frac{\partial \ell_i(q_i, c)}{\partial q_i}\frac{\mathrm{d}q_i}{\mathrm{d}\eta_i}\frac{\partial \eta_i}{\partial \beta_j}\right], \qquad j = 1, \ldots, k,
$$

where

$$
\frac{\partial \ell_i(q_i, c)}{\partial q_i} = c\,(q_i^\star - q_i^\dagger\,y_i^\star).
$$

We have that $\mathrm{d}q_i/\mathrm{d}\eta_i = 1/g'(q_i)$ and $\partial\eta_i/\partial\beta_j = x_{ij}$. Therefore, the vector $U_{\boldsymbol{\beta}} \equiv \partial\ell(\boldsymbol{\beta}, c)/\partial\boldsymbol{\beta}$ can be written in matrix notation as in Equation (12).

Differentiating (11) with respect to the parameter c leads to

$$
\frac{\partial \ell(\boldsymbol{\beta}, c)}{\partial c} = \sum_{i=1}^{n}\frac{\partial \ell_i(q_i, c)}{\partial c} = \sum_{i=1}^{n} y_i^\sharp,
$$

which leads to the second component of $\boldsymbol{U}$ given by (13).

We obtain the second-order derivatives $\ell(\boldsymbol{\beta}, c)$ with respect to $\boldsymbol{\beta}^\top$ and c, which compose the observed information matrix $\boldsymbol{J}$ from Section 5.1. For j, $p = 1, \ldots, k$, using the chain and product rules, we have

$$
\begin{aligned}
\frac{\partial^2 \ell(\boldsymbol{\beta}, c)}{\partial\beta_p\partial\beta_j} =& \sum_{i=1}^{n}\left\{\frac{\partial}{\partial q_i}\left[\frac{\partial \ell(q_i, c)}{\partial q_i}\frac{\mathrm{d}q_i}{\mathrm{d}\eta_i}\right]\frac{\mathrm{d}q_i}{\mathrm{d}\eta_i}\frac{\partial\eta_i}{\partial\beta_j}\frac{\partial\eta_i}{\partial\beta_p}\right\} \\
=& \sum_{i=1}^{n}\left[\frac{\partial^2 \ell_i(q_i, c)}{\partial q_i^2}\frac{\mathrm{d}q_i}{\mathrm{d}\eta_i} + \frac{\partial \ell_i(q_i, c)}{\partial q_i}\frac{\partial}{\partial q_i}\frac{\mathrm{d}q_i}{\mathrm{d}\eta_i}\right]\frac{\mathrm{d}q_i}{\mathrm{d}\eta_i}x_{ij}x_{ip},
\end{aligned}
$$

where $\partial^2\ell_i(q_i, c)/\partial q_i^2 = m_i + p_i\,y_i^\star$ and

$$
\frac{\partial}{\partial q_i}\frac{\mathrm{d}q_i}{\mathrm{d}\eta_i} = -\frac{g''(q_i)}{[g'(q_i)]^2}.
$$

It follows that

$$
\frac{\partial^2 \ell(\boldsymbol{\beta}, c)}{\partial\beta_p\partial\beta_j} = \sum_{i=1}^{n}\left\{\left[(m_i + p_i\,y_i^\star)\frac{1}{g'(q_i)} - c\,(q_i^\star - q_i^\dagger y_i^\star)\frac{g''(q_i)}{g'(q_i)^2}\right]\frac{1}{g'(q_i)}x_{ij}x_{ip}\right\}.
$$

In order to simplify the notation, we write $\boldsymbol{J}_{\boldsymbol{\beta}\boldsymbol{\beta}}$ in matrix form as given by Equation (14).

For obtaining the components of $\boldsymbol{J}_{c\boldsymbol{\beta}}^{\top}$ component, we set

$$\frac{\partial^2 \ell(\boldsymbol{\beta}, c)}{\partial c\, \partial \beta_j} = \frac{\partial}{\partial c}\left[\frac{\partial \ell(\boldsymbol{\beta}, c)}{\partial \beta_j}\right] = \frac{\partial}{\partial c}\left\{\sum_{i=1}^{n}\left[\frac{\partial \ell_i(q_i, c)}{\partial q_i}\frac{\mathrm{d}q_i}{\mathrm{d}\eta_i}\frac{\partial \eta_i}{\partial \beta_j}\right]\right\} = \sum_{i=1}^{n}\left[\frac{\mathrm{d}q_i}{\mathrm{d}\eta_i}\frac{\partial \eta_i}{\partial \beta_j}\right]\frac{\partial^2 \ell_i(q_i, c)}{\partial c\, \partial q_i}.$$

The right term can be written as

$$\frac{\partial^2 \ell_i(q_i, c)}{\partial c\, \partial q_i} = r_i - s_i\, y_i^{\ddagger} + u_i\, y_i^{\star}.$$

Hence, analogously to the previous calculates, we have

$$\frac{\partial^2 \ell(\boldsymbol{\beta}, c)}{\partial c\, \partial \beta_j} = \sum_{i=1}^{n}\frac{1}{g'(q_i)} x_{ij}(r_i - s_i\, y_i^{\ddagger} + u_i\, y_i^{\star}).$$

Thus, the quantity $\boldsymbol{J}_{c\boldsymbol{\beta}}^{\top}$ written in matrix notation is just given by (15).

For calculating the component J_{cc}, we have

$$\frac{\partial^2 \ell(\boldsymbol{\beta}, c)}{\partial\, c^2} = \frac{\partial}{\partial c}\left[\frac{\partial \ell(\boldsymbol{\beta}, c)}{\partial\, c}\right] = \frac{\partial}{\partial c}\left[\sum_{i=1}^{n}\frac{\partial \ell_i(q_i, c)}{\partial c}\right] = \sum_{i=1}^{n}\frac{\partial^2 \ell_i(q_i, c)}{\partial c^2}.$$

The second-order derivative of $\partial \ell_i(q_i, c)$ with respect to c is expressed as

$$\begin{aligned}
\frac{\partial^2 \ell_i(q_i, c)}{\partial c^2} = & -\frac{1}{c^2} - \frac{\log^2(\log q_i^{-1})[t(q_i) - 1]}{t(q_i)\log[t(q_i)]} + \frac{\log^2(\log q_i^{-1})\log^{2c} q_i^{-1}}{[t(q_i)]^2\log[t(q_i)]} + \frac{\log^2(\log q_i^{-1})\log^{2c} q_i^{-1}}{[t(q_i)]^2\log^2[t(q_i)]} \\
& - \frac{\log^2(\log y_i^{-1})[t(y_i) - 1]}{[t(y_i)]^2} - \frac{\log \tau^{-1}}{\log[t(q_i)]}\left[\frac{\log^2(\log y_i^{-1})\log^{c} y_i^{-1}}{t(y_i)}\right. \\
& \left. - \frac{\log^2(\log y_i^{-1})\log^{2c} y_i^{-1}}{[t(y_i)]^2}\right] + \frac{2\log(\log q_i^{-1})\log \tau^{-1}[t(q_i) - 1]\log(\log y_i^{-1})[t(y_i) - 1]}{t(q_i)t(y_i)\log^2[t(q_i)]} \\
& + \left\{\frac{\log^2(\log q_i^{-1})\log \tau^{-1}[t(q_i) - 1]}{t(q_i)\log^2[t(q_i)]} - \frac{\log^2(\log q_i^{-1})\log \tau^{-1}\log^{2c} q_i^{-1}}{[t(q_i)]^2\log^2[t(q_i)]}\right. \\
& \left. - \frac{2\log^2(\log q_i^{-1})\log \tau^{-1}\log^{2c} q_i^{-1}}{[t(q_i)]^2\log^3[t(q_i)]}\right\}\log[t(y_i)].
\end{aligned}$$

Let $y_i^{\diamond} = \partial^2 \ell_i(q_i, c)/\partial c^2$. Then, we shall define $\boldsymbol{Y}^{\diamond} = \mathrm{diag}\{y_1^{\diamond}, \ldots, y_n^{\diamond}\}$, and obtain a simpler expression for the component J_{cc}, namely

$$J_{cc} = \sum_{i=1}^{n} y_i^{\diamond} = \mathrm{tr}(\boldsymbol{Y}^{\diamond})$$

as expressed in Equation (16).
